# (Nano)biotechnological approaches in the treatment of cervical cancer: integration of engineering and biology

**DOI:** 10.3389/fimmu.2024.1461894

**Published:** 2024-09-13

**Authors:** Weimin Xie, Zhengmei Xu

**Affiliations:** Department of Gynecology, Affiliated Hengyang Hospital of Hunan Normal University & Hengyang Central Hospital, Hengyang, China

**Keywords:** cervical cancer, drug delivery, nanoparticles, hydrogels, drug resistance, tumor immunotherapy

## Abstract

Cervical cancer is one of the most malignant gynaecological tumors characterised with the aggressive behaviour of the tumor cells. In spite of the development of different strategies for the treatment of cervical cancer, the tumor cells have developed resistance to conventional therapeutics. On the other hand, nanoparticles have been recently applied for the treatment of human cancers through delivery of drugs and facilitate tumor suppression. The stimuli-sensitive nanostructures can improve the release of therapeutics at the tumor site. In the present review, the nanostructures for the treatment of cervical cancer are discussed. Nanostructures can deliver both chemotherapy drugs and natural compounds to increase anti-cancer activity and prevent drug resistance in cervical tumor. Moreover, the genetic tools such as siRNA can be delivered by nanoparticles to enhance their accumulation at tumor site. In order to enhance selectivity, the stimuli-responsive nanoparticles such as pH- and redox-responsive nanocarriers have been developed to suppress cervical tumor. Moreover, nanoparticles can induce photo-thermal and photodynamic therapy to accelerate cell death in cervical tumor. In addition, nanobiotechnology demonstrates tremendous potential in the treatment of cervical cancer, especially in the context of tumor immunotherapy. Overall, metal-, carbon-, lipid- and polymer-based nanostructures have been utilized in cervical cancer therapy. Finally, hydrogels have been developed as novel kinds of carriers to encapsulate therapeutics and improve anti-cancer activity.

## 1 Introduction

Cervical cancer is one of the main challenges for the health of females and it is among the most common tumors (1). According to the receptor of WHO in 2018, 570,000 females have been diagnosed with cervical cancer worldwide and it has caused 311,000 deaths (2–4). Moreover, the number of cervical cancer cases can increase from 570,000 to 700,000 between 2018 and 2030 (2). The incidence rate of cervical cancer is suggested to be increased in low-to-middle-income countries and in these countries, the number of deaths has been shown to be 90% of the 311,000 deaths. In high-risk countries, the incidence rate is 75 per 100,000 females, while it low-risk countries, it is 10 per 100,000 females (2, 5). There are several risk factors for the development of cervical cancer in which human papillomavirus is the most common one (2, 6). Moreover, smoking, immunosuppression, diet and socioeconomic condition can cause cervical cancer development (7–9). The treatment of cervical cancer is mainly based on irradiation, surgical resection, and chemotherapy (10). The extent of disease and the staging system (I-IV) provided by EIGO can determine the type of therapeutic modality (11, 12). For the cervical cancer patients at the early stages, the surgical resection is suggested. However, the therapy of advanced cervical cancer is challenging and requires combination therapies and application of chemotherapy (13). The platinum-based chemotherapeutics including cisplatin and paclitaxel are commonly used for the treatment of cervical cancer (13). The overall survival of cervical cancer patients has been shown to be improved upon application of chemotherapy alone or in combination with other therapies (14). Moreover, immunotherapy has been also introduced for the elimination of cervical tumor (15). However, the cervical cancer cells have been able to develop resistance into radiotherapy (16), chemotherapy (17) and immunotherapy (18). There are several reasons for the development of therapy resistance in cervical cancer such as frequent application of drugs and the dysregulation of molecular pathways. As a result, new kinds of therapeutics have been introduced for the regulation of cervical cancer progression including combination therapy and application of phytochemicals. However, these strategies have not been able to significantly improve the prognosis and survival of patients. Therefore, new kinds of therapeutic modalities should be introduced that nanoparticles are among them.

Nanomaterials are structures with size at nano-scale range with wide application from engineering to medicine. In the recent years, the biomedical application of nanoparticles has significantly improved (19, 20) and they are promising for the drug delivery (21), gene delivery (22) and diagnosis (23). In the field of oncology, the nanoparticles have been applied to improve the potential of conventional therapeutics in cancer suppression, accelerating immunotherapy, introducing new kinds of therapies such as phototherapy and improving drug delivery (24–27). This is also applicable to cervical cancer and therefore, the present review will focus on the role of nanostructures for the treatment of cervical cancer. This comprehensive review aims to illuminate the potential of nanocarriers in drug and gene delivery, the application of phototherapy, the innovative use of hydrogels as novel carriers in cervical cancer therapy, and the development of stimuli-responsive nanostructures for selective cervical cancer suppression.

## 2 Nanoparticles for the drug delivery in cervical cancer

The various kinds of chemotherapy drugs have been used for the treatment of cervical cancer. However, the recent studies have demonstrated that cervical tumor cells are able to develop resistance into conventional therapeutics such as chemotherapy (28). As a result, the researchers have focused on the introduction of phytochemicals for the treatment of cervical cancer (29–31). However, natural products often suffer from poor bioavailability, raising questions about their clinical applicability. To address this, nanostructures have been introduced to enhance drug delivery, improving their therapeutic index and efficacy in cervical cancer suppression. This section evaluates the potential of nanoparticles for optimizing drug delivery in cervical cancer therapy.

PEGylated liposomal doxorubicin was employed as salvage chemotherapy in recurrent cervical cancer; however, the patients exhibited a poor response (32). Moreover, doxorubicin side effects and/or toxicity have restricted the success of chemotherapy. Additionally, it was discovered that resveratrol has the potential to decrease the side effects caused by doxorubicin (33–35). Therefore, in the study conducted by Tomoaia and colleagues, the effects of doxorubicin combined with gold nanoparticles and resveratrol were analysed in two human cervical tumor cell lines (36). Both resveratrol-doxorubicin mixtures and doxorubicin-loaded gold nanoparticles demonstrated strong anti-cancer effects in human cervical carcinoma cells, even at a low dose of 0.1 μg/mL. Toxic effects of these therapies were first observed in HeLa and CaSki cells, with apoptosis induction validated through Annexin V-FITC/propidium iodide staining and MTT formazan cellular staining. These findings suggest that incorporating doxorubicin-loaded gold nanoparticles and resveratrol into novel drug delivery systems could be advantageous for cancer treatment (36).

Doxorubicin is widely used as a chemotherapy agent to treat many different types of cancers (37). To improve the ability of selenium nanostructures to target tumors, hyaluronic acid was added to the surface of nanostructures to create a drug delivery system known as HA-SeNPs. The functionalized nanostructures loaded with soxorubicin can be utilized to improve the effectiveness of cancer treatment in cervical carcinoma (38). These hyaluronic acid-functionalized doxorubicin-loaded nanoparticles showed preferential absorption in HeLa cells and human umbilical vein endothelial cells. They exhibited a higher rate of release in acidic settings as opposed to normal conditions, with 76.9% being released at pH 5.4. The growth of HeLa cells was effectively stopped by nanoparticles and caused cell death through the Bcl-2 pathway. It hindered the growth of tumors by inhibiting the proliferation of cancer cells and triggering apoptosis (38).

In addition to doxorubicin, selenium nanostructures are also promising for the delivery of paclitaxel in cervical cancer therapy (39). The increasing evidences have highlighted the potential of cervical cancer cells in the development of paclitaxel resistance (40, 41), emphasizing on the design of nanoparticles for its delivery. The selenium nanoparticles were examined for drug release at pH 7.4 and 5.5. These nanoparticles can deliver paclitaxel and their modification with chitosan has been performed (maybe providing their response to pH) to enhance ROS generation and impair cervical cancer progression (39). Moreover, lipid-polymer hybrid nanocarriers functionalized with folate have been developed as pH-sensitive carriers for paclitaxel delivery (42). These nanoparticles possessed particle size of 169.9 nm with size distribution of 0.151capable of suppressing cancer growth (23% ± 1.1%). In addition to reduction in tumor size, the nanoparticles did not cause weight loss in animal models (42).

**Figure 1:**
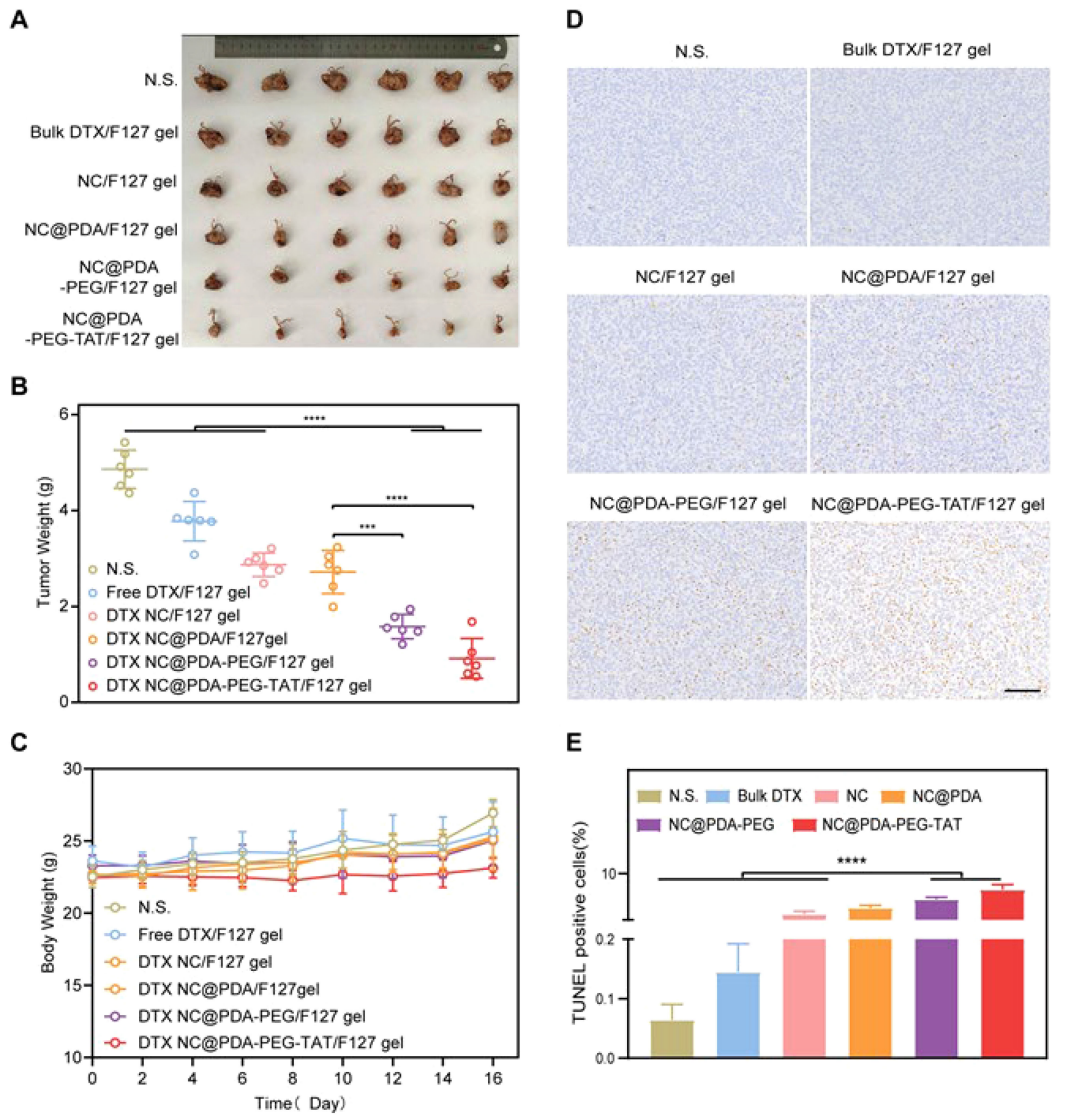
The application of nanocrystal-embedded gels upon intravaginal administration for cervical cancer therapy. **(A)** Picture of orthotropic tumors; **(B)** Tumor weight; **(C)** Body weight; **(D)** TUNEL assay; **(E)** Quantification of TUNEL assay. Reprinted with permission from Elsevier (45). *** means p<0.001, **** means p<0.0001. N.S. means Not Significant.

Another chemotherapy drug utilized for cervical cancer is docetaxel. In respect to the potential of cancer cells in the development of drug resistance, the nanocarriers have been significantly applied for the docetaxel delivery. In spite of the application of docetaxel along with other anti-cancer compounds such as melatonin (43) and schisandrin B (44), the complete elimination of tumor cells requires application of nanoparticles for docetaxel delivery. The nanocrystals are able to deliver docetaxel and they have been modified with polydopamine to mediate its conjugation with TAT peptide. The modification of nanocrystals with TAT peptide and PEG can reduce mucus entrapment of carriers. These nanoparticles are internalized in the tumor cells and can promote the anti-cancer activity of deoctaxel. Moreover, these nanoparticles have been embedded in the thermosensitive gels demonstrating high accumulation at tumor site, deep penetration at mucosa and suppression of tumor growth (**Figure 1**) (45). In addition to the cancer cells, docetaxel-loaded nanostructures are able to impair cancer stem cells (46). In order to improve anti-cancer activity, the nanoparticles have been applied for the co-delivery of docetaxel with other factors such as endostatin (47). Another approach can be application of functionalized nanoparticles with better specificity towards tumor cells. The PLGA-TPGS nanoparticles functionalized with mannitol have been developed for docetaxel delivery. The modification of nanoparticles with aptamer-polydopamine has been performed to increase targeting cervical cancer. The applied aptamer was AS1411 and these nanoparticles improved selective targeting of tumor cells and enhanced anti-cancer activity of docetaxel (48).

In addition, PAM-PBLG-b-TPGS nanoparticles have been applied for the delivery of docetaxel in cervical cancer therapy (49). PAM-PBLG-b-TPGS nanostructures displayed a number of characteristics including excellent encapsulation efficiency, small size, and favorable release characteristics. Additionally, due to the high encapsulation efficiency and controlled drug release of docetaxel-loaded PAM-PBLG-b-TPGS nanoparticles, they showed increased cytotoxicity and uptake rate in Hela cells and MCF-7 cells (49).

Cisplatin CP is a primary chemotherapy medication for treating cervical cancer, but its widespread use is hindered by the need for high doses and severe side effects. Furthermore, the recent studies have shown the development of CP resistance in cervical cancer due to the dysregulation of genetic factors including PDHB-AS and Wnt (50), VPS13C (51), HMGB1 (52) and BRSK1 (53), among others. In this regard, PEGylated liposomes have been prepared for the delivery of CP in cervical cancer therapy. The resulting nanocarriers demonstrated particle size of 97.3 nm, zeta potential of -19 mV and encapsulation efficiency of 47.7%. These nanoparticles have been prepared for targeting estrone and they can internalize in cervical tumor cells through caveolin-mediated endocytosis, exerting high anti-cancer activity (54).

**Figure 2:**
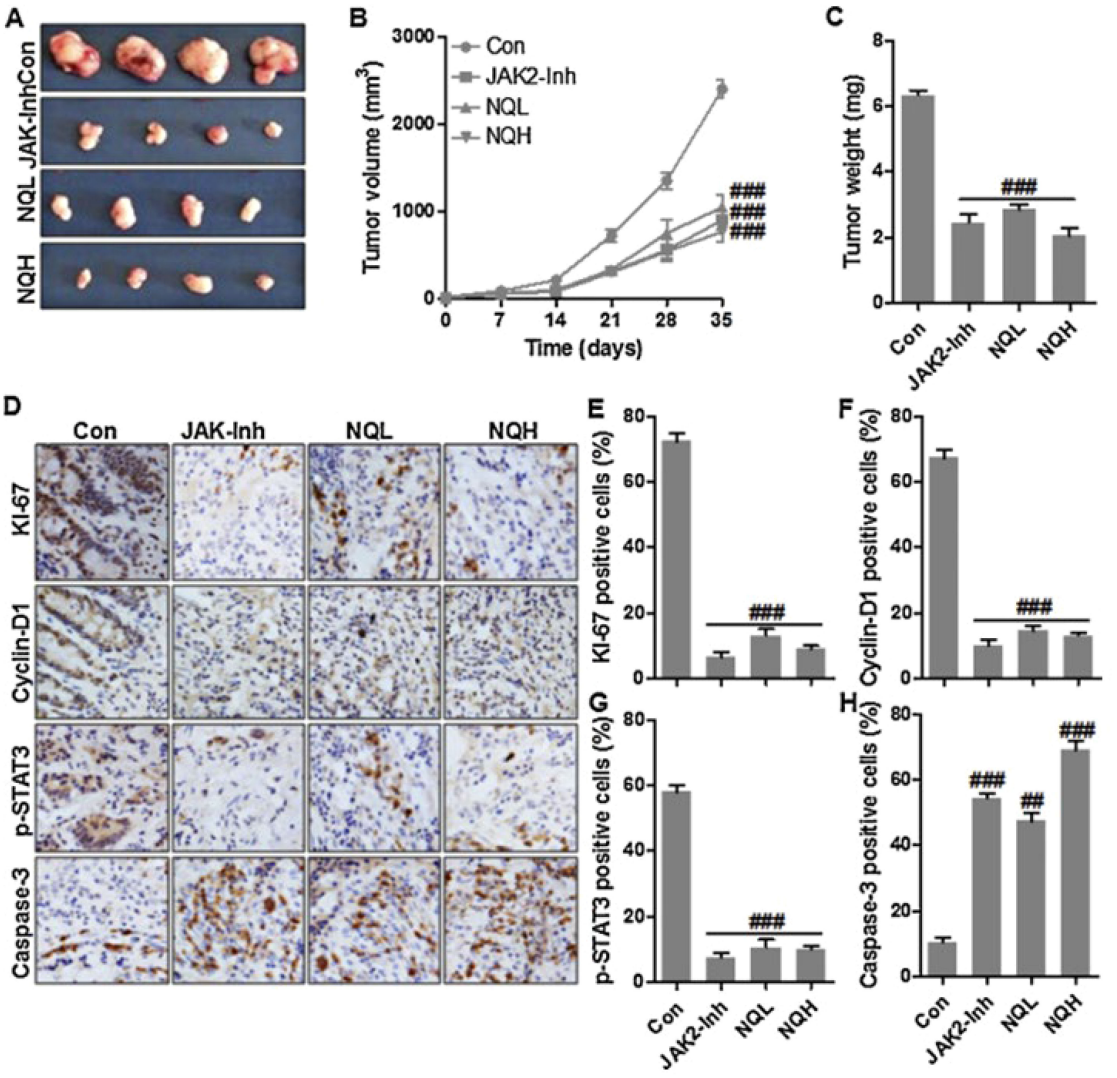
The potential of quercetin nanostructures in the treatment of cervical cancer. **(A)** Tumor size upon application of quercetin nanostructures; **(B)** Tumor volume; **(C)** Tumor weight; **(D–H)** The application of immunohistochemistry to evaluate levels of KI-67, Cyclin-D1, p-STAT3 and Caspase-3 positive cells. Reprinted with permission from Elsevier (64). ## means p<0.01, ### means p<0.001.

In addition to chemotherapy drugs used in cervical cancer and the potential of nanoparticles for their delivery, the increasing evidences have shown that phytochemicals are also promising candidates for the suppression of cervical tumor (55–57). Curcumin is one of the most commonly used natural compounds in the treatment of cervical cancer. However, its poor bioavailability limits its effectiveness. To address this issue, nanostructures have been employed to enhance its therapeutic index against cervical tumors. Specifically, a complex of curcumin and ruthenium(II) was prepared and loaded into liposomal nanostructures. These liposomes, created using a thin layer evaporation method, effectively suppressed cervical cancer (58). Notably, the curcumin can mediate the synthesis of nanoparticles. An example is the development of reduced gold nanostructures from curcumin to deliver IL-12 DNA to increase its levels in cervical cancer therapy (59). In another approach, the solid lipid nanoparticles were developed for the delivery of curcumin in cervical cancer therapy for improving its internalisation (60). The benefit of the lipid-based nanocarriers is their long-term biocompatibility, paving their way for the future clinical application. In addition to application of nanostructures for the delivery of curcumin in cervical cancer therapy (61, 62), other kinds of bioactive compounds have been also delivered. In line with this, hydroxyapatite nanostructures have shown potential in the delivery of quercetin capable of cargo release in response to pH. The quercetin-loaded nanoparticles increase ROS generation and decrease the proliferation of cervical tumor cells (63). One of the limitations of current studies is the poor attention to the underlying mechanisms affected in cervical cancer therapy. The quercetin nanostructures have been shown to suppress JAK2 and induce apoptosis and autophagy in cervical cancer therapy (**Figure 2**) (64). However, autophagy has a dual function in human cancers and more studies are required to highlight autophagy control (inhibition or induction) by nanoparticles in cervical cancer therapy.

Current studies highlight a promising approach for drug delivery in cervical cancer therapy through the use of nanoparticles. Both natural products and synthetic drugs have been effectively delivered using these nanostructures, demonstrating their potential for cervical tumor suppression. First of all, it is suggested for the studies to provide more information regarding the co-delivery of natural products and synthetic drugs for the suppression of cervical cancer and reversing chemoresistance. Furthermore, special attention should be given to the co-delivery of natural products and genetic tools to enhance tumor suppression efficacy. The potential of metal and carbon nanoparticles in drug delivery for cervical cancer warrants further investigation. Additionally, several specific mechanisms, including dysregulation of autophagy, are evident in cervical cancer and require exploration (65, 66) and ferroptosis (67, 68). Therefore, the specific regulators of autophagy and ferroptosis should be loaded on nanoparticles for the regulation of tumorigenesis in cervical cancer. Moreover, the dual function of autophagy should be also considered. Nanostructures can overcome drug efflux mechanisms by inhibiting the function of P-glycoprotein (P-gp), preventing it from pumping anticancer drugs out of cancer cells, thereby increasing the accumulation of drugs within cancer cells (69). Additionally, nanocarriers can enhance endocytosis, allowing more drugs to enter the interior of cancer cells, thereby increasing efficacy (70, 71). In modifying the tumor microenvironment, nanocarriers can release drugs under the acidic conditions of the tumor microenvironment, enhancing the targeting and effectiveness of the drugs, or by regulating the redox reactions within tumor cells, increasing the activity of anticancer drugs and reducing drug resistance (72, 73). Photothermal and photodynamic therapies are also important applications of nanostructures. Through near-infrared light irradiation, certain nanostructures (such as gold nanorods) can generate localized high temperatures, destroying cancer cells and increasing drug sensitivity; while photodynamic therapy uses nanocarriers to deliver photosensitizers, which generate reactive oxygen species under specific wavelength light irradiation, directly killing cancer cells and enhancing the anticancer effect (74). The role of nanoparticles in bypassing the function of P-glycoprotein as drug efflux transporter or overcoming drug resistance requires more investigation.

## 3 Nanoparticles for the gene delivery in cervical cancer

In response to the development of drug resistance, research has focused on exploring new strategies for cancer therapy and enhancing tumor cell response to chemotherapy. Gene therapy has emerged as a novel approach for treating human cancers, designed to target specific genes or regulate particular molecular pathways. By addressing factors involved in drug resistance, gene therapy can be tailored to target these mechanisms. However, gene therapy faces challenges such as off-target effects and poor accumulation at tumor sites. To overcome these issues, nanoparticles have been developed to deliver genetic factors effectively, impair tumorigenesis, protect genetic materials from degradation, and improve accumulation at tumor cells and tissues. This approach also helps to reduce off-target effects.

The highly specific gene-silencing ability of small interfering RNA (siRNA) makes it a promising new option for therapy. Nevertheless, for advancing new therapeutic approaches, it is necessary to have effective and secure delivery services. Therefore, creating effective delivery systems for siRNA is essential for advancing siRNA-based cancer treatments. In this regard, biocompatible selenium nanoparticles have been functionalized with RGD peptide and then, loaded with Derlin1-siRNA in cancer therapy. These nanoparticles demonstrated high cellular uptake in HeLa cells using clathrin-mediated endocytosis and gene silencing decreased the proliferation, metastasis and induced apoptosis. Moreover, these nanoparticles enhanced ROS production and decreased mitochondrial membrane potential in cervical cancer therapy (75). The genes can be delivered along with chemotherapy drugs in cervical cancer suppression. Notably, the lipid nanoparticles can deliver siRNA regulating HPV16 E6/E7 along with application with cisplatin in cervical cancer therapy. These nanoparticles and cisplatin can suppress tumor growth and they downregulate HPV16 E6/E7, while they upregulate p21, p53 and HLA class I proteins. The *in vivo* highlighted the potential of these nanocarriers in enhancing cell death and reducing tumor growth. The expression of HPV16 E6/E7 decreased up to 80% by the nanoparticles (76). However, the future studies can focus on the co-delivery of drug and siRNA in synergistic cervical cancer therapy.

In addition, the nanoparticles have been applied for the delivery of siRNA targeting E7 and MCL-1 in cervical cancer therapy (77). Researchers investigated the efficacy of a cocktail of siRNA targeting oncoproteins E6, E7, and MCL-1 for treating HPV-induced lesions. The combination of siRNA anti-E7 and anti-MCL-1 showed high efficacy on HPV cell lines without affecting healthy cells. Vaginal administration was considered due to its potential in treating female reproductive tract diseases. PEGylated lipoplexes were designed to protect and deliver the siRNA to the cervico/vaginal epithelium. The nanovector complexed with active siRNA efficiently reduced mRNA levels *in vitro* and demonstrated proper properties for diffusion in mucin networks. *In vivo* experiments in mice showed sustained coverage of mucosal epithelium, highlighting the potential of PEGylated lipoplexes for delivering active siRNA for HPV treatment (77).

Furthermore, Ma and colleagues revealed that poly (β-Amino Ester) nanoparticles along with HPV16-targeting CRISPR/shRNA have the potential to serve as medications for HPV16-Related cervical cancer (78). The goal of this research was to create nanoparticles using poly (β-amino ester) (PBAE) and CRISPR/short hairpin RNA (shRNA) targeting HPV16 E7, with the purpose of decreasing HPV16 E7 levels as a potential treatment for HPV infection and associated cervical cancer. The nanomaterials exhibited minimal toxicity in both cells and mouse organs. These nanoparticles were able to hinder the growth of cervical cancer cells and xenograft tumors in nude mice, as well as revert the malignant cervical epithelium phenotype in HPV16 transgenic mice by decreasing the expression of HPV16 E7. The shRNA-loaded nanomaterials demonstrated superior performance compared to CRISPR-loaded nanostructures. Potential drugs for treating HPV infection and HPV-related cervical malignancy could be created by combining PBAE and CRISPR/shRNA in HPV-targeting nanocarriers (78).

In another research, Nunes and colleagues examined alteration of chitosan-TPP nanoparticle characteristics for delivery of plasmid DNA vaccines (79). The findings indicated that the nanomaterials had a round/oval form, a small size range < 180 nm, and high zeta potentials (>20 mV), as well as strong durability after a month of storage at 4°C in the formulation buffer or when exposed to culture medium and trypsin. Removing formulation buffers improved the cell viability rate in nanoparticle cytotoxicity experiments. The E7 antigen transcription was also elevated in nanostructures produced with a high pDNA concentration of 60 μg/mL. The examined CS-TPP-pDNA polyplexes show potential as an effective method for nucleic acid vaccines, not only for combating viral infections, but also for addressing new and future pathogens (79). Therefore, the accumulating data demonstrate that nanoparticles are potential carriers for the delivery of genetic tools in cervical cancer therapy.

In spite of the significant focus on the application of nanoparticles for the gene delivery and good promise, there are still a number of limitations that can be considered for the development of better and novel nanostructures for gene delivery in cervical cancer therapy. Most of the studies have focused on the application of nanostructures for siRNA delivery in cervical cancer therapy. However, more focus should be paid on the delivery of shRNA, CRISPR/Cas9 system and plasmids in cervical cancer therapy. Moreover, the studies should focus on the development of more functionalized nanostructures in gene delivery. It is suggested to functionalize nanoparticles with peptides and others to increase targeting the cervical tumor cells and reduce off-targeting activity. The different kinds of nanostructures have been utilized for the gene delivery, but a focus should be made on stimuli-responsive nanoparticles. Moreover, more focus should be made on hydrogels and carbon-based materials for gene delivery in cervical cancer therapy.

In nanotechnology-based cervical cancer treatment, individual genetic, molecular, and physiological differences among patients significantly impact the efficacy and safety of therapies (80). Variations in genetic mutations and drug-metabolizing enzymes can affect the metabolism and effectiveness of nanomedicines, while molecular features and biomarkers of tumors determine the targeting and efficacy of these therapies (30). Additionally, patients’ physiological conditions (such as body weight, body fat, and blood flow dynamics) can influence drug distribution and accumulation, and differences in immune systems may affect the immune recognition and clearance of nanocarriers (81). Personalized treatment can be achieved by identifying drug-response-related mutations through genomic sequencing, selecting the most suitable nanomedicines based on biomarker detection, and adjusting drug dosages and administration based on the patient’s physiological state (82). This personalized approach, considering genetic, molecular, and physiological factors, enhances the targeting and effectiveness of nanotechnology therapies, reduces side effects, and optimizes treatment outcomes, ultimately improving the overall patient experience.

## 4 Stimuli-responsive nanoparticles in cervical cancer therapy

In the previous section, it was shown that nanostructures can deliver drugs and genes in cervical cancer therapy. In order to better explore the potential of nanocarriers in cancer therapy, the stimuli-responsive nanostructures have been developed. The stimuli-responsive nanostructures have been developed for responding to the specific stimuli in tumor microenvironment (TME) including pH, redox status, enzymes, temperature and hypoxia to accelerate the release of cargo at tumor site. Therefore, the recent studies have significantly focused on the application of stimuli-responsive nanocarriers in cancer therapy (83–87). The TME is a dynamic and complex environment comprised of tumor cells, stromal cells, fibroblasts, immune cells, blood vessels, extracellular matrix and signalling molecules capable of regulating tumorigenesis and therapy resistance. These specific features can be utilized for the development of stimuli-responsive nanoparticles. The pH-responsive nanoparticles can be developed based on application of materials including poly(β-amino esters), poly(L-histidine) or modification with pH-sensitive moieties. The most commonly developed nanostructures for the treatment of cancer is pH-sensitive nanocarriers (88, 89). The redox-sensitive nanocarriers can be developed based the disulfide cross-linkers or tuiolated polymers and they have been also used for the drug delivery and immunotherapy in human cancers (90, 91). The enzyme-responsive nanoparticles can be developed based on the enzymes present in the TME such as metalloproteinases (92). Thermo- and hypoxia-sensitive nanoparticles are other kinds of nanostructures utilized in cancer therapy that can respectively respond to temperature and hypoxia. In order to develop hypoxia-sensitive nanoparticles, the linkers sensitive to hypoxia or the molecules such as nitroimidazoles can be utilized. For the thermo-sensitive nanostructures, the materials including poly(N-isopropylacrylamide) (PNIPAM) can be utilized. Both thermo- and hypoxia-responsive nanomaterials have been also significantly utilized in cancer therapy (93–96). The next parts discuss the application of stimuli-responsive nanoparticles in cervical cancer therapy.

Surface modified nanoparticles decorated with specific ligands can be targeted to organs, tissues, cells, or cellular organelles, affecting their interactions and *in vivo* efficacy (97, 98). Folate (FA) is a promising ligand for targeted anticancer drug delivery, as it targets cancer cells with high affinity for folate receptors over-expressed in various human carcinomas (99–102). In this respect, a dual treatment approach for cervical cancer involving lipid-polymer hybrid nanoparticles loaded with carboplatin and paclitaxel was designed, which is pH-sensitive and decorated with folate (42). The dimensions of FA-CBP/PTX-LPNs were found to be 169.9 ± 5.6 nm, exhibiting a tight size uniformity of 0.151 ± 0.023. FA-carboplatin/pacltiaxel-lipid nanoparticles showed drug release that responds to pH, efficient uptake by cells (66.7 ± 3.1%), and strong ability to inhibit cell growth (23 ± 1.1%). The highest tumor distribution and tumor inhibition effectiveness of nanostructures was observed *in vivo*, with no noticeable weight loss. The FA-carboplatin/pacltiaxel-lipid nanomaterials have proven to be a promising treatment for cervical cancer due to their effective antitumor results in both *in vitro* and *in vivo* models with widespread tumor distribution (42).

In nanotechnology-mediated cancer treatments, tumor cells can develop resistance through various mechanisms. Firstly, factors within the tumor microenvironment, such as acidic conditions and hypoxia, may affect the stability and cellular uptake of nanomedicines, reducing their effective concentration within cancer cells (103). Additionally, an immunosuppressive environment may enhance the clearance of nanomedicines or alter their targets. Tumor cells can also reduce nanomedicine uptake and increase drug efflux by modifying cellular uptake mechanisms or overexpressing drug efflux pumps (such as P-glycoprotein (P-gp)) (104). Changes in the cell membrane and membrane proteins can further affect the binding of nanoparticles. At the molecular level, tumor cells might activate resistance-related genes (such as multidrug resistance genes (MDR)), enhance antioxidant responses, or alter key signaling pathways (such as PI3K/Akt and MAPK) to diminish the efficacy of nanomedicines (105, 106). Strategies to overcome these resistance mechanisms include optimizing nanoparticle design, combining with other therapeutic approaches, targeting resistance mechanisms, and employing personalized treatment strategies. These methods can enhance the therapeutic effectiveness of nanotechnology-based therapies and reduce resistance, thereby fully realizing their potential in cancer treatment.

**Figure 3:**
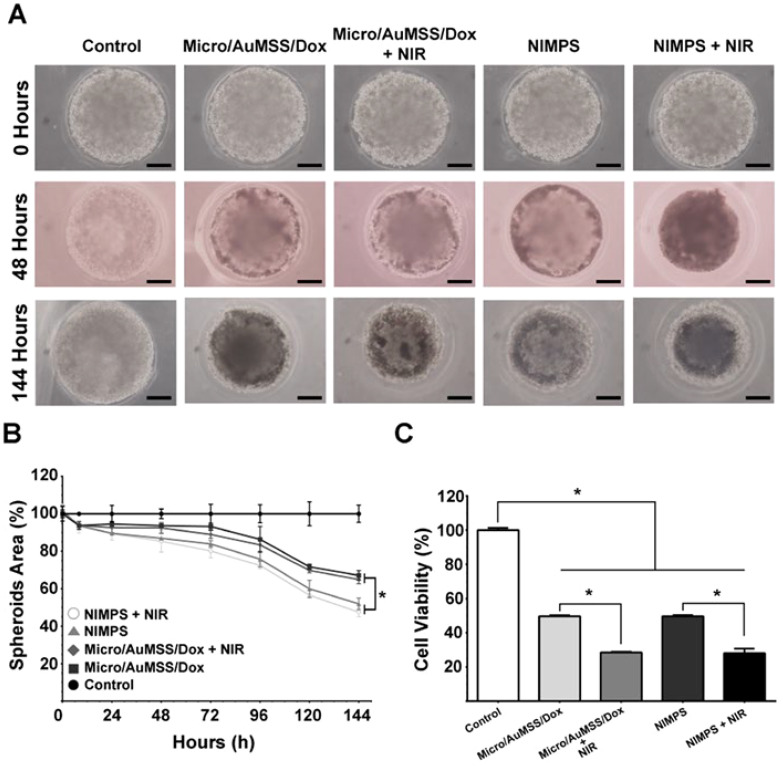
Highlighting the anti-cancer activity of NIMPS. **(A)** The changes in the 3D HeLa spheroids area after incubation with microspheres; **(B)** Cytotoxicity characterization. **(C)** Cell viability after treatment. Reprinted with permission from Elsevier (107). * means p<0.05.

Moreover, combination drug therapy is becoming increasingly recognized in the scientific community as an effective method to enhance treatment effectiveness and advance the eradication of cancer. Hence, In this line, a novel pH- and thermo-responsive carrier was developed through merging doxorubicin-filled gold-core silica shell nanorods with poly(lactic-co-glycolic acid) based microparticles loaded with salicylic acid (NIMPS) (107). The results indicated that the release of drugs and nanorods could be activated by either near-infrared (NIR) laser exposure or exposure to an acidic environment. The 2D cell studies conducted *in vitro* demonstrated that the NIMPS are well-tolerated by HeLa cells and readily absorbed by them. Furthermore, the use of 3D cell culture models showed that when NIMPS administration was combined with NIR laser irradiation, it could decrease the size of HeLa spheroids by up to 48% (**Figure 3**) (107).

Furthermore, Chen and colleagues researched a pH-responsive liposome modified with comb-like pseudopeptides that anchor to the membrane, to improve intracellular delivery and treat cancer (108). Based on results, pseudopeptides that possess endosomolytic characteristics have a strong affinity for liposomal membranes, enhancing cellular absorption. Virus-like systems responsive to pH can regulate the release of content by modifying the type and quantity of polymer used. Multifunctional liposomes can disrupt endosomes and effectively transport doxorubicin to cancer cells. Liposomal systems containing doxorubicin that resemble viruses are more successful in targeting different cancer cells than plain liposomes or doxorubicin alone. These findings indicate that cervical cancer treatment could be enhanced by improving cytoplasmic transport (108).

In another research, lim and colleagues investigated how redox-responsive gold nanoparticles coated with HA and FA can be used for targeting anticancer therapy (109). Based on the findings, methotrexate (MTX) was continuously delivered in glutathione (GSH). The experiments on cellular intake demonstrated that FA-HA-ss-gold nanostructure was more efficiently taken up than HA-ss-gold nanostructure in the inner parts of the tumor. Additionally, the release experiments offered compelling proof that FA-HA-ss-gold nanocarriers function as carriers that respond to GSH. Tests in laboratory conditions demonstrated that FA-HA-ss-gold/MTX nanocarriers had superior anti-tumor effects on HeLa and BT-20 cancer cells compared to gold only and HA-ss-gold/MTX nanomaterials, with no harm to HEK-293 cells (109).

**Figure 4:**
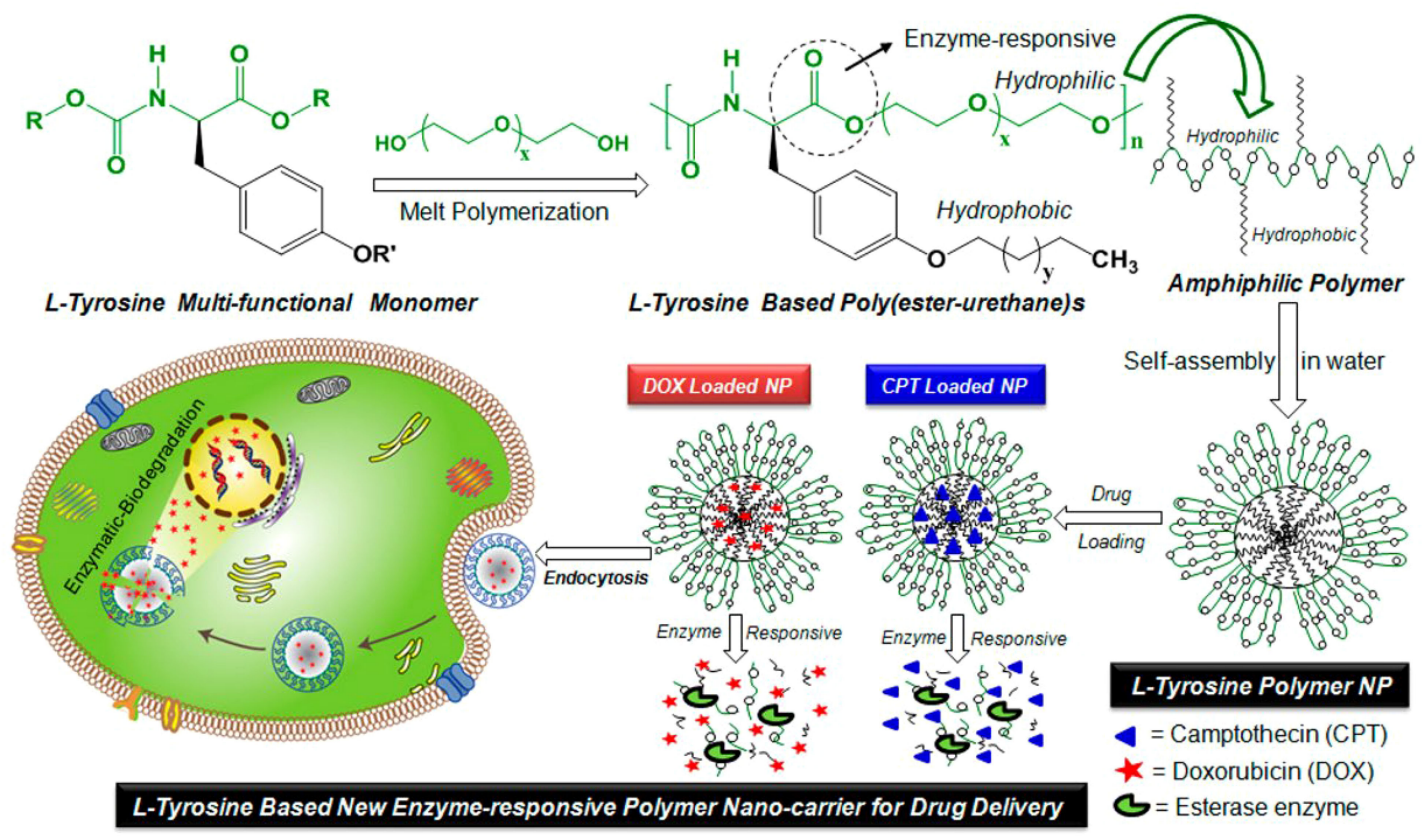
The enzyme-responsive nanoparticles have been developed using l-tyrosine based amphiphilic poly(ester-urethane)s for the treatment of cervical cancer through delivery of doxorubicin and camptothecin. Reprinted with permission from ACS (110).

Furthermore, Aluri and his team researched the growth of l-tyrosine enzyme-responsive amphiphilic poly(ester-urethane) nanocarriers for delivering multiple drugs to HeLa cervical cancer cells (110). Amphiphilic polymers self-assembled into 200 ± 10 nm nanoparticles with high encapsulation capabilities for anticancer drugs DOX and CPT. Drug release studies showed stability at extracellular conditions and enzymatic biodegradation at intracellular level. Cytotoxicity tests on HeLa and WT-MEFs cell lines demonstrated non-toxicity of l-tyrosine nanoparticles and effectiveness of drug-loaded nanoparticles in cancer cell killing. Confocal imaging confirmed cellular uptake, with higher drug uptake from nanoparticles than free drugs. Synthesis of poly(ester-urethane)s and enzyme-responsive drug delivery explored new possibilities for l-tyrosine materials in various applications (**Figure 4**) (110).

The Current studies underscore the potential of stimuli-responsive nanocarriers in cervical cancer treatment. While ensuring biocompatibility and long-term safety, research should also focus on enhancing the specificity and selectivity of these nanostructures for targeting specific organelles and cells within the tumor microenvironment (TME). Despite the advancements in multifunctional stimuli-responsive nanoparticles, their scalability and feasibility for large-scale production need thorough evaluation at the clinical level. Several materials, such as poly(β-amino esters) and poly(L-histidine), have been underexplored in the development of stimuli-responsive nanoparticles for cervical cancer therapy. Given that the redox status is often disrupted in the TME, there is a need for focused research on redox-responsive or combined redox- and pH-responsive materials. PNIPAM is a well-known material for thermo-sensitive nanoparticles in cervical cancer therapy. The potential of nitroimidazole and L-tyrosine-based poly(ester-urethane) for developing hypoxia- and enzyme-sensitive nanoparticles should also be evaluated. Comb-like pseudopeptides, which enhance membrane binding and intracellular accumulation, show promise for the development of pH-sensitive nanostructures. Additionally, combining PLGA nanoparticles with gold-core silica shell nanorods could lead to the creation of multifunctional nanostructures responsive to both pH and near-infrared (NIR) stimuli.

## 5 Nanoparticles in cervical cancer phototherapy

### 5.1 Photodynamic therapy

Nanoarchitecture-accelerated phototherapy is a promising therapeutic approach for the cancer therapy mainly including photothermal (PTT) and photodynamic (PDT) therapy. In PDT, the nanoparticles are able to deliver photosensitizes to enhance the generation of reactive oxygen species (ROS) for mediating cellular damage and death. However, in nanoparticle-mediated PTT, nanoparticles convert light into heat for the induction of hyperthermia to cause cell death. In the recent years, nanoparticle-mediated phototherapy has been used for the suppression of various human cancers. The cancer metastasis can be suppressed through nanostructure-induced PTT. The human serum albumin nanostructures deliver indocyanine green to induce PTT in ablation of cancer metastasis and provide cancer imaging (111). In breast cancer, PLGA nanoparticles have been used to co-deliver IR792 and alpha-tocopherol, with a particle size of 80.4 nm, to suppress metastasis and induce oxidative damage by increasing ROS levels (112). Noteworthy, the nanoparticle-mediated phototherapy has been shown to boost cancer immunotherapy (113, 114). In this section, the potential of nanoparticles for mediating PDT and PTT in cervical cancer therapy is evaluated.

Despite being a promising photosensitizer for PDT of cancer, there is limited information on the impact of HYP-induced PDT on cervical cancer, with most data coming from studies conducted on the HeLa cell line (115–118). Moreover, the antiproliferative effect of HYP has not been studied in cell lines originating from squamous cervical cancer cells (SiHa, CasKi, and C33A), which represent the predominant type of cervical cancer globally (accounting for 75%-85% of all cases) (119, 120). Consolaro and team investigated the targeted photodynamic impact on cervical cancer cells using P123 Pluronic^®^-based nanoparticles to control hypericin distribution (121). HYP/P123 micelles exhibited successful and specific phototoxic effects on cervical cancer cells in a time- and dose-dependent manner, while HaCaT cells were unaffected. Additionally, HYP/P123 micelles were found to gather in endoplasmic reticulum, mitochondria, and lysosomes, leading to PDT-accelerated cell death primarily through necrosis. HYP/P123 caused oxidative stress in cells primarily through the type II mechanism of PDT and blocked cancer cell movement and invasion mainly by inhibiting MMP-2 (121).

Combining multiple therapies into a single platform has the potential to address the limitations of traditional single therapy methods and enhance the effectiveness of cancer treatment. Consequently, polymeric nanoparticles known as PPHE have been designed through self-assembly and encapsulation process, consisting of Pheo a, EAE7 targeting ligand, γ-PGA, MPEG-PLGA, and HA, to improve the treatment of HPV-positive cervical cancer using PDT/CAP combination therapy and increase its effectiveness (122). PPHE polymeric nanoparticles, with an average size of 80.5 ± 17.6 nm, demonstrated better PDT effectiveness on CaSki cells because of their improved targeting capability. The combination of PDT/CAP successfully stopped the growth of cervical cancer cells by raising reactive oxygen species levels and triggering apoptosis. A 3D cell culture model verified the powerful treatment potential of PPHE-based PDT/CAP combination therapy on CaSki cells, suggesting it as a highly successful approach for treating cervical cancer (122).

Recently, there has been a significant increase in simultaneous PDT/PTT therapy to enhance the therapeutic effect (123–125). Methylene blue (MB) is sensitive to light and has become a common photosensitizer utilized in combination with nanoparticles for treating cancer (126–128). Due to the high efficiency of singlet oxygen species in boosting the photolysis of multiple cancer cell lines, MB has been incorporated into different therapeutic nanoplatforms (129, 130). Kuo and colleagues was conducted on creating Core-Shell Nanoparticles made of anisotropic cu ferrite-polymer for the purpose of Photodynamic Ablation of Cervical Cancer Cells (131). The study developed methylene blue-immobilized copper-iron (MB-CuFe) nanoparticles through a one-step hydrothermal reaction to enhance phototherapeutic effects. Control over the Fe/Cu ratio was achieved by adjusting the iron precursor concentration. The CuFe nanomaterials acted as a Fenton catalyst, converting hydrogen peroxide into reactive oxygen species and showed potential for magnetic resonance imaging (MRI). The FDA-approved MB photosensitizer adsorbed strongly onto the CuFe NPs, aiding in drug delivery and improving photodynamic therapy at 660 nm. Low cytotoxicity was observed in cervical cancer cells (HeLa cells). CuFe NPs were found to be degradable in an acidic environment, reducing long-term retention risks. The high photo-to-thermal conversion of CuFe NPs could be utilized in combination with photodynamic therapy for effective cancer cell depletion with deep-red light irradiation (131).

Furthermore, Shah and colleagues in their research conducted on PEGylated doped- and undoped-TiO2 nanoparticles for photodynamic therapy of cervical cancers (132). PEG nanocarriers decreased the survival of HeLa cells when exposed to solar and UV radiation. Adding cobalt and nitrogen to TiO2 nanocrystals enhanced their ability to activate light in the visible/near-infrared spectrum, yet undoped-TiO2 that was PEGylated was better at exterminating cancer cells. PEGylated undoped-TiO2 made using sol-gel process exhibited notable photodynamic impact, resulting in 75% death of HeLa cells at 5.5 μg/mL concentrations when exposed to UV or sunlight. These results indicate that PEGylated TiO2 nanocarriers may offer potential as a viable choice for photodynamic therapy when treating cervical cancer (132).

Furthermore, in their study, Benito and colleagues examined how the combination of 5-aminolevulinic acid and gold nanoparticles can work together to improve photodynamic therapy for cervical cancer (133). Research was conducted on the increased production of protoporphyrin IX (PpIX) in cancer cells through the use of 5-aminolevulinic acid (ALA). Comparison between ALA and ALA combined with gold nanoparticles (ALA-AuNPs) was conducted for PDT on cervical cancer cells. ALA-Au nanomaterials raised the generation of ROS, boosting phototoxicity. Two different sized Au nanomaterials (14 nm and 136 nm) were created and examined with ALA for ROS generation, cellular viability, and cellular apoptosis. ALA-Au nanomaterials triggered cell death through ROS following PDT. Combining ALA with Au nanomaterials resulted in increased cytotoxicity and cell damage compared to using ALA alone or ALA combined with Au nanomaterials (133).

In another investigation conducted by Vega and colleagues the role of porphyrin-based polysilsesquioxane nanoparticles in photodynamic therapy of cervical cancer cells was examined (134). TCPP silane derivatives were used in a reverse microemulsion technique to produce polysilsesquioxane nanoparticles that can respond to redox reactions. The release of TCPP was demonstrated by a redox-responsive mechanism using a reducing agent. TCPP-PSiQ nanomaterials showed internalization in cancer cells. *In vitro* photoxicity tests revealed that the TCPP-PSilQ nanomaterials that respond to redox showed a better phototherapy effect on cervical cancer cells than a non-responsive TCPP-PSilQNP control material (134).

### 5.2 Photothermal therapy

IR-820, a novel form of ICG, is seen as a hopeful theranostic agent among commonly utilized organic photothermal agents (135–139). Nevertheless, enhancing the biocompatibility of IR-820 is essential for broadening its scope in biomedical applications. Encapsulation of polymers is a desirable technique to improve biocompatibility. Polymers, when used as enclosure matrices, have the ability to shield molecules from outside disturbances (140). Hence, utilizing polymer-coated IR-820 is an effective method for improving biomedical uses (141–143). The PTT for cervical cancer cells is enhanced with the use of IR-820 nanoparticles coated with a polymer that emits near-infrared light (144). IR-820@PSMA nanomaterials demonstrated exceptional photothermal stability and biocompatibility. The NIR fluorescent confocal microscopic imaging technique was used to confirm the cellular uptake of the IR-820@PSMA nanomaterials in HeLa cells. The IR-820@PSMA nanoparticles were used in the PTT of living HeLa cells with a 793 nm laser, resulting in a PTT efficiency of 73.3% (144).

Creating nanoparticles that can trigger the generation of ROS is now a crucial tactic in cancer treatment. At the same time, it is essential to create multifunctional nanoparticles that can react to the TME for effectively diagnosing and treating tumors. Thereefore, GSH-responsive MoS2@MnO2 theranostic nanoparticles have been prepared for multimodal imaging and photothermal/chemodynamic therapy of cervical cancer (145). The nanoparticles were additionally altered with Methoxypoly(Ethylene Glycol) 2000 (mPEG-NH2) to enhance their biocompatibility, leading to the creation of MoS2@MnO2-PEG. These nanoparticles were found to exhibit notable imaging capabilities for MRI and CT, which can be beneficial for tumor diagnosis. MoS2@MnO2-PEG has the ability to fight against tumors, causing a notable reduction in tumor cell numbers when used in combination treatment. These nanoparticles show promising potential for antitumor therapy through the combination of CDT/PTT and can be further investigated in the field of biomedical research (145).

**Figure 5:**
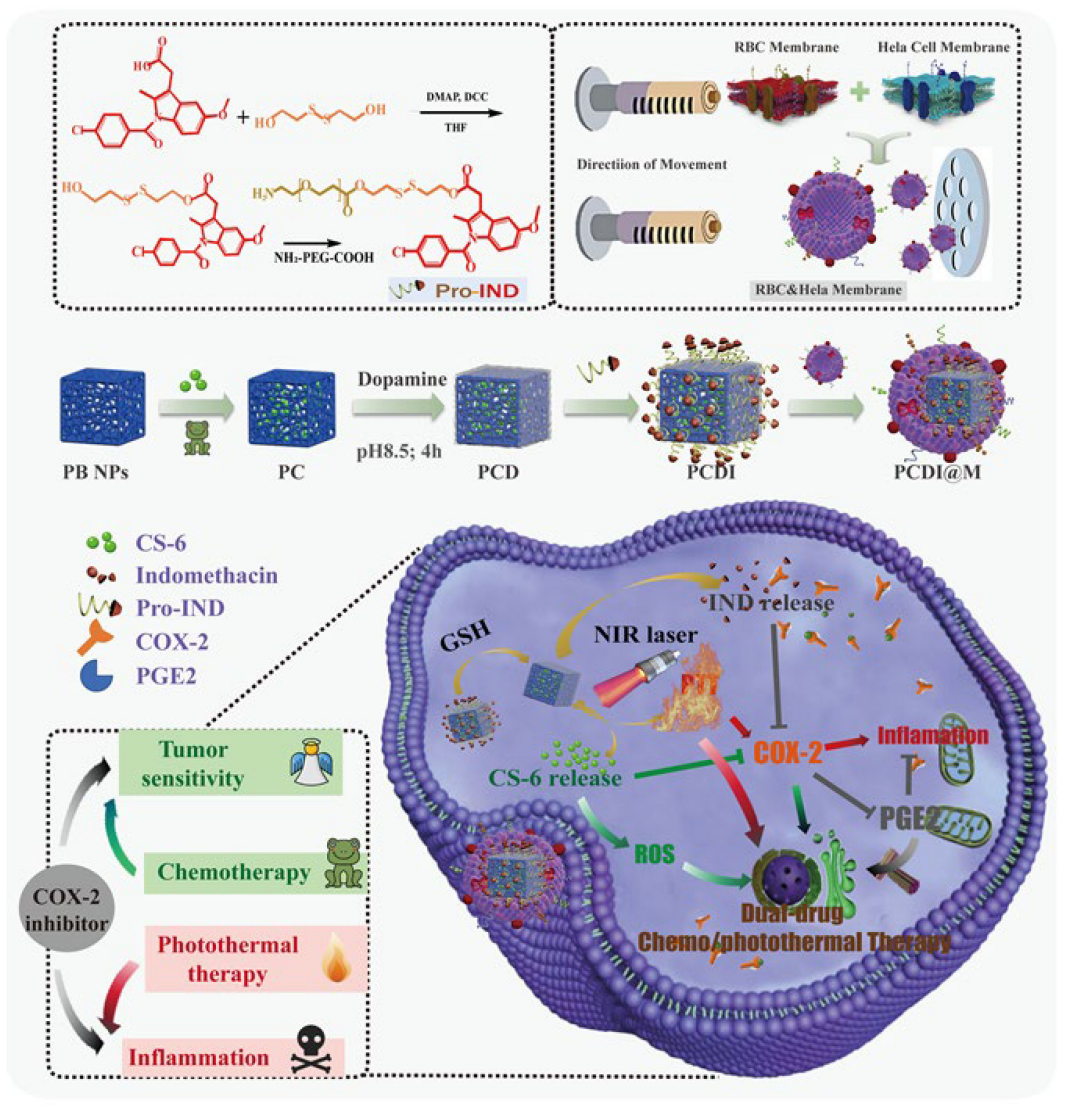
The preparation of the PCDI@M nanoparticles and the synergistic impact occurring between chemotherapy and PTT. These biomimetic nanoparticles developed from the membrane of red blood cells were able to release IND upon NIR irradiation for the downregulation of COX-2. Moreover, the increased ROS levels. Reprinted with permission from Elsevier (146).

The biomimetic nanoparticles loaded with gamabutolin-indomethacin for treating cervical cancer through a combination of chemotherapy and PTT, as well as for reducing inflammation (146). This study presents the use of pro-nanodrug complexes that respond to GSH and NIR, enhancing gamabufotalin-induced chemotherapy/PTT through reprogramming the TME with indomethacin. Additionally, a hybrid cell membrane was utilized to provide nanocomplexes with an extended circulation time and increased drug accumulation in tumor tissue. Indomethacin, when activated by elevated GSH levels, can reduce tumor inflammation caused by PTT and make tumor cells more sensitive to gamabufotalin by stopping the secretion of PGE2. The low-dose gamabufotalin that has been released has minimal side effects and is able to effectively destroy tumor cells by producing ROS and reducing COX-2 expression. The nanocomplexes were highly effective in treating tumors in mice. This was seen by the complete removal of cervical tumors and a notable increase in the survival time of the mice when using chemo-photothermal therapy (**Figure 5**) (146).

Furthermore, the polymer-coated ICG nanoparticles can be utilized for improved PTT and near-infrared fluorescence imaging in cervical cancer (147). ICG was packaged inside nanoparticles through the use of the amphiphilic polymer poly(styrene-co-maleic anhydride) (PSMA) to create ICG@PSMA nanoparticles. Additionally, a thorough analysis was conducted on the optical and thermal properties of ICG@PSMA nanoparticles. Additionally, the positive biocompatibility of ICG@PSMA nanoparticles was shown in cell viability experiments on a human cervical cancer cell line (HeLa). Therefore, ICG@PSMA nanoparticles were also utilized to improve PTT of live HeLa cells with 808 nm excitation, resulting in achieving a PTT efficiency of approximately 70%. The potential of using ICG nanoparticles as a PTT nanoplatform in the novel could lead to enhanced options for treating tumours in the future (147).

Moreover, the functionalized magnetic nanoparticles have shown the potential to be utilized for NIR-induced PTT, which can be applied in the treatment of cervical cancer (148). The FPI nanomaterials exhibited round shapes with even dispersal and high chemical durability. By exposing FPI nanomaterials to a 793 nm laser, they were able to produce a temperature increase of 54.1°C and achieve a photothermal conversion efficiency of 35.21%. Further assessment and verification on HeLa cells with a high survival rate (90%) confirmed the minimal toxicity of FPI nanomaterials. Additionally, when exposed to laser radiation at 793 nm, FPI nanoparticles displayed efficient PTT properties for HeLa cells. As a result, FPI nanomaterials, being a promising PTA, hold significant potential in the realm of PTT for cancer therapy (148).

In addition, the blue hydrogel nanoparticles facilitate PTT on cervical cancer cells (149). Hydrogel nanoparticles containing Coomassie Brilliant Blue G dye embedded in their polyacrylamide structure have proven effective in inducing thermolysis in the immortalized human cervical cancer cell line (HeLa) across various concentrations and treatment durations. These versatile particles have previously been utilized in cancer research for tumor boundary identification, glioma surgery, and photoacoustic imaging. Incorporating photothermal therapy (PTT) into these nanoparticles offers a three-pronged approach to cancer treatment, integrating guided tumor surgery with intraoperative photoacoustic imaging and intraoperative PTT (149).

Overall, the current studies provide the significance of the nanoparticles for the induction of PTT and PDT in cervical cancer therapy. However, a number of limitations and perspective as following should be considered in the future studies. The studies have mainly focused on the application of polymeric nanocarriers for cervical cancer phototherapy. In spite of the biocompatibility of the polymeric nanocarriers, their long-term safety, their degradation into monomers, the metabolism and the approaches for decreasing their toxicity (maybe through green synthesis methods) can improve their application in cervical cancer therapy. Although the nanoparticle-mediated phototherapy is promising in cervical cancer therapy, the scalability of this approach at the clinical level requires investigation. Therefore, the clinical studies are required to highlight its potential and also, the strategies for the large-scale production of these nanocarriers. Moreover, the nanoparticle-mediated phototherapy has been beneficial for the induction of immunogenic cell death and TME remodelling that has been ignored in cervical cancer. Finally, the phototherapy can also induce autophagy that in case of protective function, the autophagy suppression can promote nanoparticle-accelerated phototherapy in cervical cancer suppression.

## 6 An overview of different nanoparticles for cervical cancer therapy

### 6.1 Polymeric-based nanoparticles

The polymeric nanostructures have been also extensively in cancer therapy and they can be prepared from biocompatible and biodegradable polymers. They are promising candidates in drug delivery to improve therapeutic index and reduce the adverse impacts. The commonly used polymers for the synthesis of polymeric nanostructures are PLGAm PEG and PLA polymers that are biocompatible and have been significantly used for drug delivery in cancer therapy. These nanocarriers have been also used for the treatment of cervical cancer (150, 151). Notably, the polymeric nanoparticles are able to release drug in a sustained manner and they can be functionalized with ligands and antibodies in cancer therapy. Polymeric nanostructures have a small size at the range of 10-200 nm and through mediating EPR, they can enhance the accumulation at the tumor site. In addition, polymeric nanoparticles can mediate immunotherapy (152) and phototherapy (153). Therefore, the current section focuses on the application of polymeric nanocarriers in cervical cancer therapy.

Focused drug delivery is a sought-after treatment method in cancer nanotechnology, involving the transportation of medications to specific body parts (154–156). The cell-penetrating peptide (CPP) trans-activating transcriptional activator (TAT) is frequently utilized for cell entry in both natural and altered forms (157). Utilizing TAT for drug delivery can enhance drug activity and penetration, leading to more effective treatment, reduced side effects, and overcoming drug resistance (158, 159). Thus, Liu and colleagues studied the combined delivery of paclitaxel and TOS-cisplatin using solid lipid nanoparticles (SNLs) targeted with TAT, showing synergistic antitumor effects on cervical cancer (160). Based on results, heLa cells successfully internalized TAT PTX/TOS-CDDP SLNs and exhibited a combined effect in inhibiting cervical tumor cell proliferation. *In vivo*, they showed high accumulation in tumor tissue, excellent effectiveness against tumors, and significantly reduced toxicity. The current research suggests that the co-delivery method shows potential as a combined treatment for cervical cancer, and potentially other kinds of cancer too (160).

SLNs are widely utilized for targeted drug delivery with enhanced bioavailability and decreased toxicity. Therefore, a different method was utilized to deliver SLNs containing 5-flourouracil (5-FU) within thermo-sonic nano-organogel (TNO) variants for targeted stimuli-responsive treatment of cervical cancer. Nanospherical SLNs containing PLA, palmitic acid (PA), and polyvinyl alcohol (PVA) were developed and added to TNO variants with additional thermal and ultrasound triggers to release 5-FU in the cervix while maintaining pharmaceutical stability (161). SLNs were able to achieve rate-modulated release of 5-FU within an organogel when exposed to either single (thermo-) or combined (thermo-sonic) stimuli. The release of 5-FU from all TNO variants exhibited an initial burst on day 1 followed by sustained release over 14 days. TNO 1 showed the most desirable release over 15 days, releasing 44.29% under single stimuli and 67.13% under combined stimuli. The release rates were influenced by the SLN: TO ratio, biodegradation, and hydrodynamic influx. TNO 1 (1:5) released 46.8% of 5-FU by day 7, similar to its initial mass, compared to other TNO variants. The TNO variants may be used as a platform for site-specific delivery of chemotherapeutic agents for treating cervical cancer (161).

In a different study, the potential anticancer properties of aspirin and polymeric micelles linked to 2’-hydroxy-2,3,5’-trimethoxychalcone were investigated for their ability to induce apoptosis in cervical cancer (162). The nanomaterials exhibited an IC_50_ value of 4.172 µM in HeLa cells. Additionally, HeLa cell proliferation was significantly inhibited and apoptosis was stimulated by 10 µM NPs. Moreover, lipid accumulation in HeLa cells caused by nanomaterials resulted in the induction of apoptosis and mitochondrial dysfunction, triggering apoptotic. Collectively, the findings of this study show that the developed nanoparticles induced apoptosis by enhancing lipid accumulation in HeLa cells, indicating a potential new approach to enhancing the effectiveness of CVC cancer treatment (162).

Furthermore, another experiment evaluated the healing capabilities of new curcumin-filled TPGS/F127/P123 mixed polymeric micelles (Cur@NPT100) for treating cervical cancer (163). The Cur@NPT100 nanoparticles possessed an average of 19 nm in size, had a zeta potential of -4 mV, a drug loading of 8.18%, and an encapsulation efficiency of 79.38%. They remained unchanged in water and gradually released medication over a period of 6 days. During cellular experiments, the NPT100 nanoparticles enhanced the absorption of Cur in cancerous cells compared to healthy cells, resulting in elevated cell mortality and a stoppage in the cell cycle. The nanoparticles also raised Cur levels in mitochondria and interfered with mitochondrial function, causing apoptosis. In cell and animal experiments, Cur@NPT100 demonstrated better tumor-fighting abilities and compatibility than free Cur, indicating its potential use in treating cervical cancer (163).

In another research, Li and colleagues investigated how to make paclitaxel-loaded and FA-modifiedPLGA nano-micelles and tested their anticancer impact on cervical cancer HeLa cells (164). Nano-micelles were fine-tuned to have a particle size of (125.3 ± 1.2) nm, PDI of 0.086 ± 0.026, zeta potential of (-20.0 ± 3.8) mV, drug loading of 7.2% ± 0.75%, and encapsulation efficiency of 50.7% ± 1.0%. They displayed a round form when observed under TEM. Empty FA-PLGA-NMs had little impact on the growth of tumor cells, whereas nano-micelles loaded with drugs and PTX in its free form displayed considerable inhibition. PTX@FA-PLGA-NMs showed an IC_50_ of 0.56 μg·mL and blocked cell migration. They triggered programmed cell death in HeLa cells and improved cell absorption, indicating promise for treating cervical cancer. These findings suggest that PTX@FA-PLGA-NMs show potential as effective nano-drug transporters for enhanced tumor treatment (164).

Moreover, Niu and colleagues conducted a study exploring controlled drug delivery for cervical cancer chemotherapy using polylactide stereocomplex micelle (165). An effective treatment for cervical carcinoma was created by developing a steady drug delivery system that utilizes doxorubicin-loaded stereocomplex micelles made from enantiomeric poly(ethylene glycol)-poly(D-lactide) and poly(ethylene glycol)-poly(L-lactide). The micelles containing doxorubicin (PDM/DOX, PLM/DOX, SCM/DOX) had a size of approximately 100 nm, taking advantage of the EPR effect. SCM/DOX exhibited the most sluggish release of the drug, the greatest absorption by tumor cells, and the most effective suppression of tumor cells *in vitro*, as well as high rates of inhibiting tumor growth in a mouse model of cervical carcinoma. The micelles decreased overall toxicity of doxorubicin, offering potential as a drug delivery method for treating cervical carcinoma in the future (165).

Furthermore, in their study, Zhang and colleagues investigated the non-invasive delivery of Ce6 and doxorubicin in liposomes responsive to NIR light for combined treatment of cervical cancer (166). Nano-drug delivery system increases drug concentration in tumors by leveraging enhanced permeability and retention effect, reducing harm to healthy tissues. Ce6 emits ROS upon NIR laser exposure, causing drug release in tumor cells. This method allows for effective merging of chemotherapy and PDT, leading to substantial inhibition of tumor growth. The research emphasizes the capacity of this delivery method for various drug solubilities and successful cancer treatment (166).

Besides, Afshar-Kharghan reported a recent finding that CD59 is overexpressed in cervical cancers and other cancers, but has low expression in normal cells (167). CD59 acts as a regulatory element in cell membranes and enhances the ability of tumor cells to evade the immune system (168). In this respect, CD59 receptor targeted delivery of miRNA-1284 and CP-loaded liposomes for effective therapeutic efficacy against cervical cancer cells (169). In HeLa cells, CD59 antibody-conjugated LP-miCP (CD/LP-miCP) demonstrated a notably greater cytotoxic effect when compared to miRNA-1284/CDDP-loaded liposomes (LP-miCP). miR-1284 exhibited a characteristic dose-dependent cytotoxic effect on cervical cancer cells as a result of reducing HMGB1 levels. CD/LP-miCP achieved the highest apoptosis rate (~ 60%) among the treatments tested, surpassing both CP (~ 20%) and miR-1284 (~ 12%) treatments, demonstrating superior anticancer efficacy in cancer cells. Significantly, CD/LP-miCP increased the duration of drug presence in the bloodstream in rats with AUC(0-t) of CD/LP-miCP being 6.9 times higher than that of free CDDP. In the same way, CD/LP-miCP displayed a decrease in clearance by a factor of eight and a t1/2 3.6 times higher than that of free CP. In general, the findings showed that combining therapeutic components in a targeted and synergistic manner could be effective in treating cervical cancer (169).

### 6.2 Metal-based nanoparticles

Gold nanoparticles are difficult to synthesize in the NIR region with large amounts of organic solvents (170). Silica is popular in biomedicine due to stability, low immunogenicity, and biocompatibility, making it an ideal material for coating (171). Gold nanoparticles on modified SiO2 particles are used for PTT (172). Thus, silica-coated gold nanoparticles were developed and attached to antibodies targeting SR-BI for potential use in tracking and treating cervical cancer. The Au@SiO2-SR-BI antibody was created with fluorescein isothiocyanate (FITC) labeling and then subjected to characterization analysis (173). Au@SiO2 nanoparticles had a size range of 2-2.5 μm with uniform distribution. MS751 cells expressed high levels of SR-BI in the cytomembrane. FITC-Au@SiO2-SR-BI nanoparticles had higher cell surface presence compared to FITC-Au@SiO2. Treatment with FITC-Au@SiO2-SR-BI induced significant apoptosis in MS751 and H8 cells. Photothermal ablation of solid tumors was achieved with 808 nm wave activation. Antibody-conjugated Au@SiO2 nanoparticles targeted cancer cell receptors for potential cervical cancer therapy (173).

Furthermore, curcumin-modified gold nanoparticles enhance the delivery of IL-12 to an *in vitro* cell model of cervical cancer (59). Results indicate that stable, spherical AuNPs efficiently condensed and shielded the IL-12 DNA while showing good tolerance *in vitro*. The positive characteristics of this AuNP delivery system and the substantial IL-12 expression achieved suggest promising prospects for cytokine-based therapy or immunotherapy in cervical cancer (59).

Gold-coated magnetic nanoparticles offer a promising magnetic targeting strategy for clinical application in cervical cancer treatment (174, 175). These nanoparticles allow for improved accumulation at tumor sites using a magnetic field, providing effective localized therapy (176). The core-shell structure of gold-coated magnetic nanoparticles offers stabilization, biocompatibility, and surface reactivity, making them advantageous agents (177–179). While they have been widely used in detection and biomolecule immobilization, their potential as radiosensitizers for cervical cancer treatment has not been fully explored. Thereby, magnetic gold nanoparticles with a core-shell structure (Fe₃O₄@Au) have been utilized to enhance the combined effects of radio-photothermal therapy for cervical cancer (180). The Fe₃O₄@Au nanoparticles showed excellent plasmon resonance, superparamagnetic behavior, compatibility with living organisms, and efficiency in converting light into heat. Low levels of nanoparticles and short exposure to NIR light resulted in the death of cervical cancer cells. The synergy of anti-cancer effects was achieved by combining radiotherapy and PTT. Enhanced NP absorption and effectiveness were achieved with the help of an external magnetic field. Fe₃O₄@Au nanoparticles exhibit potential as nanoagents for precise treatment of cervical cancer (180).

The therapeutic potential of silver nanoparticles in cervical carcinoma cells varies with time and concentration (181). Silver nanostructure exposure of HeLa cells caused cell death and programmed cell death. Cytotoxic effects were shown by the SRB assay. Microscopic examinations showed morphological alterations suggesting apoptosis. DNA fragmentation confirmed the presence of necrosis and apoptosis. Elevated levels of ROS and over-expression of p53, bax, and caspase 3 mRNA were detected. Activity of the enzyme caspase-3 was observed to have an increase as well. Silver nanostructures show promise for treating cervical cancer (181).

Although biodegradable electrospun materials offer advantages in regulating fiber morphology, porosity, and composition, the quick drug release remains a significant disadvantage (182, 183). There is a current trend towards using composite drug delivery fibers with nano- and microparticles to improve drug retention and achieve sustained release (184–186). Electrospinning is a perfect technique for producing multifunctional fibers of different sizes by utilizing polymer blends and colloidal solutions (187–190). As a result, embedding doxorubicin and iron oxide nanocubes together in polycaprolactone fibers has been shown to merge magneto-thermal and chemotherapeutic impacts on cancer cells (191). Integrating nanocubes with a size of 23 nm into fibers resulted in formations that mimic the chain-like structures found in magnetosomes of magnetotactic bacteria. These magnetic fibers showed heat-generating properties comparable to magnetosomes and were compatible with fibroblast cells without requiring chemotherapy or magnetic hyperthermia. Nonetheless, nanocube-infused fibers with doxorubicin exhibited cytotoxicity against cervical cancer cells through magnetic hyperthermia. These experiments were conducted in clinical magnetic hyperthermia settings, demonstrating that the combination of hyperthermia-induced heat damage and improved doxorubicin diffusion at therapeutic temperatures resulted in a more efficient approach to treating cancer (191).

Besides, coreshell super paramagnetic iron oxide nanoparticles (SPIONs) have been explored for delivering curcumin in cervical cancer (192). The chemical co-precipitation method was used to develop SPIONs. The nanoparticles were then modified with SDS, filled with curcumin, and covered with chitosan to form a core-shell structure. The coreshell particles produced were approximately 40 to 45 nm in size and were used for drug delivery experiments on HeLa cells, a cervical cancer cell line. It was observed that the coreshell SPIONs released curcumin between 6 and 12 hours, as shown by higher levels of apoptotic cells and increased caspase 3 expression in HeLa cells (192).

M. dioica is a medicinal plant containing various bioactive compounds like phenolic, flavonoid, carotenoid, and glycosides (193). The entire M. dioica plant has been traditionally used as a remedy for bacterial infections, malaria, inflammation, diabetes, boosting the immune system, and as an antiseptic, among other uses (194). Therefore, the potential of Momordica dioica mediated gold nanoparticles in inducing both extrinsic and intrinsic apoptosis in cervical cancer cells was investigated (195). The developed silver nanostructures were evenly distributed, had a crystalline structure, had a negative surface charge of -23.9 mV, and were spherical particles with an average size of 9.4 nm. Furthermore, the silver nanocarriers remained stable in buffer solutions and were also found to be compatible with normal human cells, such as human vascular endothelial cells and human lung cells. The silver nanostructures showed anticancer effects on various types of cancer cells including human breast cancer cells, human cervical cancer cells (HeLa), and human lung cancer cells. Additionally, in HeLa cells compared to untreated cells, the expression of pro-apoptotic genes like Bcl2 decreased while BAX, Caspase-3, -8, and -9 increased. The silver nanostructures triggered apoptosis by increasing the presence of ROS inside the cells. This is believed to be the initial study on creating bioactive metal nanoparticles from M. dioica, which has the potential to introduce new possibilities in medical treatments (195).

### 6.3 Carbon-based nanoparticles

The carbon-based nanomaterials have been significantly applied in the treatment of cancer (196, 197) owing to their excellent characteristics including high surface area, electrical and thermal conductivity and ease of functionalization. There are different kinds of carbon-based nanomaterials including carbon dots, fullerenes and carbon nanotubes. In the current section, the ability of carbon-based materials in the treatment of cervical cancer is followed.

MTX is effective against solid tumors like cervical cancer but can cause cardiotoxic effects like cardiomyopathy and reversible heart failure (198, 199). However, its efficiency in treating larger tumors via ethanol administration is limited (200). To enhance the effectiveness of MTN treatment and minimize its side effects, we developed a targeted drug delivery system using carboxylated graphene quantum dots modified with NH2-PEG-NH2 and FA. This system exhibited potent anti-tumor effects in live subjects (201). A successful drug delivery system was created using FA-PEG-cGQDs, achieving optimal entrapment efficiency (97.5%) and drug-loading capacity (40.1%). The nanosystem primarily entered human cervical cancer cells through a pathway that depended on macropinocytosis. There is remarkable anti-cancer effectiveness and minimal harm to the body’s overall system of this nanodrug distribution method (201).

Graphene oxide (GO) is structure that has been oxidized. Up to now, GO has primarily been examined for its role in transporting drugs, rather than as a treatment by itself for diseases such as glioblastoma or cervical cancer. Nevertheless, Grodzik and the team suggested an encouraging fresh method by utilizing GO to enhance the effectiveness of CP chemotherapy. They examined how triple GO pretreatment, followed by CP treatment, impacted cancerous cell lines U87 and HeLa, as well as the noncancerous cell line HS-5 (202). GO treatment increased U87 and HeLa cell sensitivity to CP, reducing viability to 40% and 72%, respectively, with no effect on HS-5 cells. There were no changes in live cells with GO pretreatment, but GO-pretreated HeLa cells had double the decrease in viability compared to CP alone. U87 cells had 2.5 times more LDH release with GO, while HS-5 cells did not (202).

**Figure 6:**
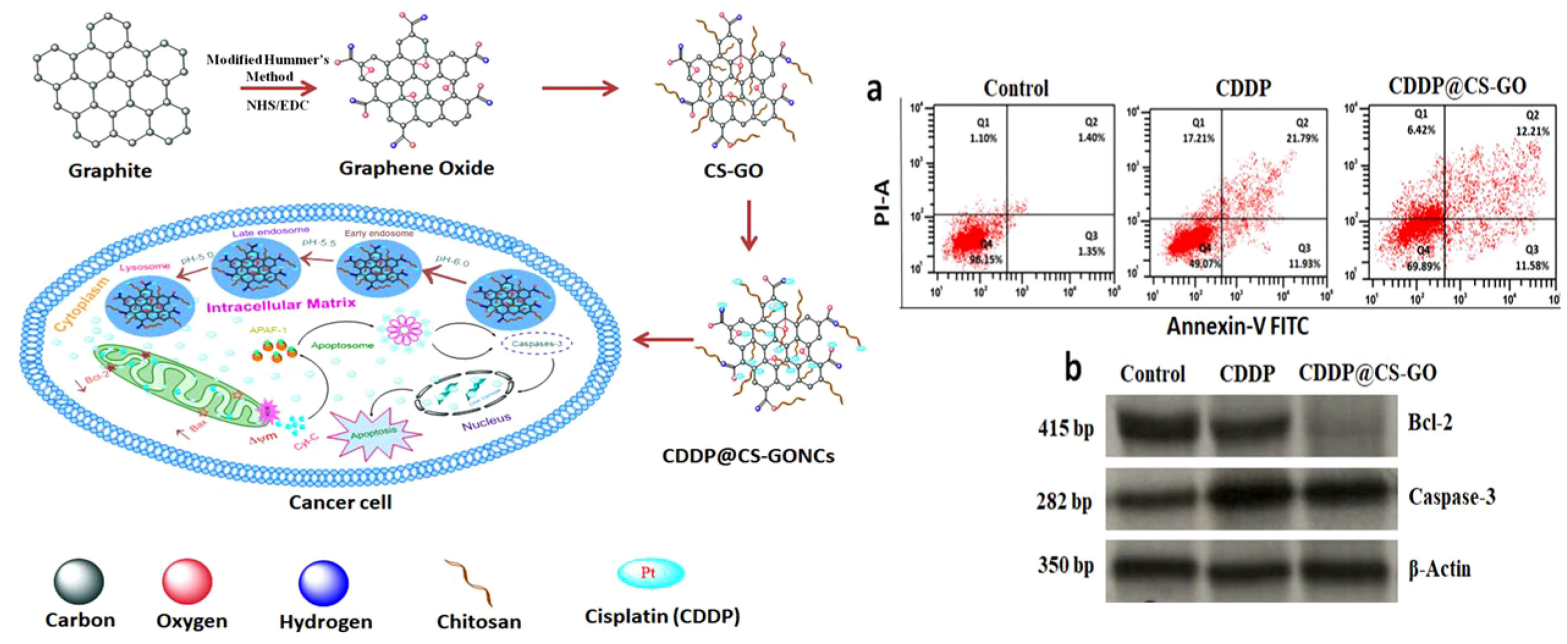
Left side) The development of nanomaterials and their pathway for the reduction of tumorigenesis, Right side) The stimulation of apoptosis by CP@CS-GO nanostructures; a) The analysis by flow cytometery, b) Evaluating protein levels of caspase-3 and Bcl-2. Reprinted with permission from ACS (203).

Moreover, there has been investigation using nanodrug delivery systemusing a CP-loaded chitosan-functionalized GO nanocomposite (CDDP@CS-GO NC). The design of multifunctional nanocomposites made from bio-graphene allows for the delivery of drugs inside cells for treating cervical cancer (203). CP@CS-GO NCs result in a notable enhancement of drug buildup inside tumor cells. Cancer cells absorb the nanocomposite through endocytosis and produce ROS inside the cell to enhance loss of mitochondrial membrane potential (Δψm), leading to the release of cytochrome C, displacement of Bcl-2 into the cytosol, and triggering of caspase-3 activation for induction of cancer cell apoptosis (**Figure 6**) (203).

Furthermore, carbon-based materials can be used for the diagnostic aims and also, during surgery of cervical tumor. In line with this, the detection of sentinel lymph node during laparoscopic surgery for cervical cancer of the uterus with carbon nanoparticles was investigated (204). The total and two-sided detection rate was 93.3% (42 out of 45) and 60.0% (27 out of 45), respectively. An increased body mass index was linked to a lower rate of detecting on both sides (P = .015). 225 SLNs were collected, averaging 5.0 ± 3.6 per patient. 81.3% of SLNs were found in anticipated areas like external iliac (39.1%), internal iliac (25.8%), and obturator (16.4%) regions. The remaining 18.7% were in unexpected locations such as common iliac (10.7%), parametrial (7.6%), and presarcal (0.4%) regions. No positive lymph nodes were discovered in non-SLNs, resulting in a 0% false-negative rate. Using CNPs, laparoscopic SLN mapping seems to be straightforward and effective for early-stage cervical cancer patients (204).

Moreover, structurally organized mesoporous carbon nanoparticles (MCN) have the potential to serve as a transmembrane delivery carrier in human cancer cells (205). MCN has a high inhibitory concentration (IC_50_) value (>50 microg/mL per million cells) demonstrating its decent biocompatibility *in vitro*. MCN substance has the ability to act as a carrier across the cell membrane, allowing Fura-2 to enter HeLa cells and release molecules within them (205).

Bee venom’s main component, melittin (MEL), shows promise as a cancer treatment despite stability challenges. In this respect, GO-based magnetic nanocomposites (PEG-GO-Fe_3_O_4_) have been used as drug carriers for MEL, investigating the impact of PEG-GO-Fe3O4/MEL complexes on human cervical cancer HeLa cells (206). PEG-GO-Fe_3_O_4_ displayed a range of distinctive physical and chemical characteristics that led to various interactions with MEL, ultimately leading to the release of MEL. PEG-GO-Fe_3_O_4_/MEL not only significantly increased the inhibitory impact on HeLa cells but also triggered the creation of pores in the cell membrane, resulting in cell lysis. PEGylated GO in this emerging drug delivery system functions as a protector for MEL, while Fe_3_O_4_ nanoparticles serve as magnetic responders. This enables the sustained release of active MEL for up to 72 h, effectively maintaining its inhibitory effect on HeLa cells (206).

Furthermore, GO-silver nanoparticle nanocomposites and CP working together increase apoptosis and autophagy in human cervical cancer cells (207). The silver nanostructures that were produced were evenly spread out, consistent, and round in shape, with an average diameter of 10 nm and evenly placed on graphene layers. Cisplatin, GO, rGO, silver nanoparticles, and rGO-silver nanoparticles caused a decrease in cell viability depending on the concentration. The combination of CP and rGO-silver nanostructures had a marked impact on cell proliferation, cytotoxicity, apoptosis, and gene expression. They stimulated autophagosomes, autophagolysosomes, and ROS, indicating promise as a complementary treatment for cervical cancer alongside CP or other chemotherapy drugs (207). Therefore, the present studies highlight that various kinds of nanocarriers can be utilized in the treatment of cervical cancer. In spite of the fact the all of them demonstrate significant toxicity on cervical cancer, the clinical application of these structures depends on their biocompatibility and therefore, the application of lipid-based nanocarriers is suggested in this case.

## 7 Hydrogels in cervical cancer therapy

The new emerging structures for the treatment of cancer are hydrogels as three-dimensional networks with polymeric structure capable of water absorption. Hydrogels have high biocompatibility and they can mimic the extracellular matrix. In the recent years, the application of hydrogels for the treatment of cervical cancer has increased and they have been significantly used for drug delivery (208), gene delivery (209), diagnosis (210), chemotherapy (211), phototherapy (212, 213), immunotherapy (214) and vaccine development (215). The current section will focus on the application of hydrogels in cervical cancer therapy.

A new product known as TraceIT hydrogel (TH; Augmenix, Waltham, MA), which is a unique iodinated polyethylene glycol hydrogel that offers visibility across different imaging modalities for a duration of 3 months, was recently released. This specific hydrogel material has been evaluated for its use as a marker in cervical cancer brachytherapy, but has not yet been evaluated as a spacer in clinical trials involving gynecologic cancers (216). Therefore, Damato and his team’s goal was to assess the impact of placing a tube between the cervix, rectum, and bladder in female cadavers, in comparison to the usual method of using gauze packing, in order to protect organs at risk during the planning of radiation therapy for cervical cancer (217). The hydrogel was effectively inserted to separate the bladder and rectum from the cervix in all five cadavers. The spacer was readily recognizable on both CT and MRI scans. Using hydrogel along with packing led to a 22% reduction in rectum D2cc dose (p = 0.02), a 10% decrease in bladder D2cc (p = 0.27), and no alteration in sigmoid D2cc dose. There was no observed distinction between using only hydrogel and using only gauze packing (217).

Poxolamer-based thermosensitive hydrogel is used in gynecology for vaginal infections, health, hygiene, and contraception. It transitions from liquid to gel form with body heat (218–221). Thermosensitive hydrogels are better than conventional ones for vaginal drug delivery due to less leakage, sustained delivery, and ease of use (222). No reports yet on using it to prevent cervical cancer recurrence. The delivery of carboplatin through the vagina using a thermosensitive hydrogel has been performed to hinder local recurrence of cervical cancer in mice (223). This research created a model of cervical/vaginal cancer recurrence mimicking surgery by injecting U14 murine cervical cancer cells into the vaginal submucosa, followed by drug treatment 24 h later to prevent tumor formation/recurrence. Injecting fluorescein sodium-loaded hydrogel into mice’s vaginas led to a high concentration of fluorescein sodium in the vagina with minimal detection in other organs. The carboplatin-loaded poloxamer hydrogel showed excellent effectiveness and overall safety in preventing the formation/recurrence of cervical cancer in mice (223).

Moreover, hydrogels that produce oxygen can help in treating tumors by improving photodynamic/gas therapy effectiveness in the presence of low oxygen levels (224). The gel within the system can partially penetrate water to interact with CaO2 and produce continuous oxygen through the catalase-like function of HCePA. The system effectively relieved hypoxia in TME for a duration of 7 days, aligning with the “inject once, irradiate again” approach and improving PDT effectiveness. Additionally, during the PDT process, the produced singlet oxygen (_1_O_2_) can also convert l-Arg into elevated levels of nitric oxide for combined gas therapy. The new oxygen supplied and drug delivery Gel system is an innovative approach for synergistic PDT/gas therapy in the treatment of cervical cancer (224).

Among women, cervical cancer is the fourth most common, and treating the vaginal mucosa locally with therapeutic agents has proven to be effective. Thermosensitive gels, originally utilized for birth control and treating infections, are now being investigated for cancer with the addition of therapeutic nanoparticles. They can be 3D printed as well to enhance the anatomical fit, ultimately improving the effectiveness of local delivery treatments. Therefore, a focused delivery approach using a Pluronic F127-alginate hydrogel was designed that has effective nanoparticle release capabilities for intravaginal treatments (225). The study showcased the degradation kinetics and nanoparticle releasing abilities of a hydrogel using ultrasmall gold nanoparticles (80% released in 48 h). The mucoadhesive properties were tested, showing 19% mucin adsorption. The hydrogel’s potential for 3D printing was demonstrated with high geometrical precision (225).

Furthermore, the injectable hydrogels have been also introduced for the treatment of cervical cancer. The injecting hydrogel spacer in the meso-sigmoid can help protect the sigmoid colon during cervical cancer brachytherapy (226). The dose ratio of sigmoid colon D2cc to HR-CTV D90 was 1.03, 0.43, 0.56, and 0.47 in different brachytherapy sessions, suggesting an increase in dose to HR-CTV while maintaining a safe sigmoid dose by using a hydrogel spacer in the meso-sigmoid. Additionally, the substance stayed in position for an extended period, and the protective qualities of the spacer persisted. During HDR-ISBT treatment, the hydrogel spacer in the meso-sigmoid region allows for a higher dose to the tumor while limiting exposure to the sigmoid colon (226).

**Figure 7:**
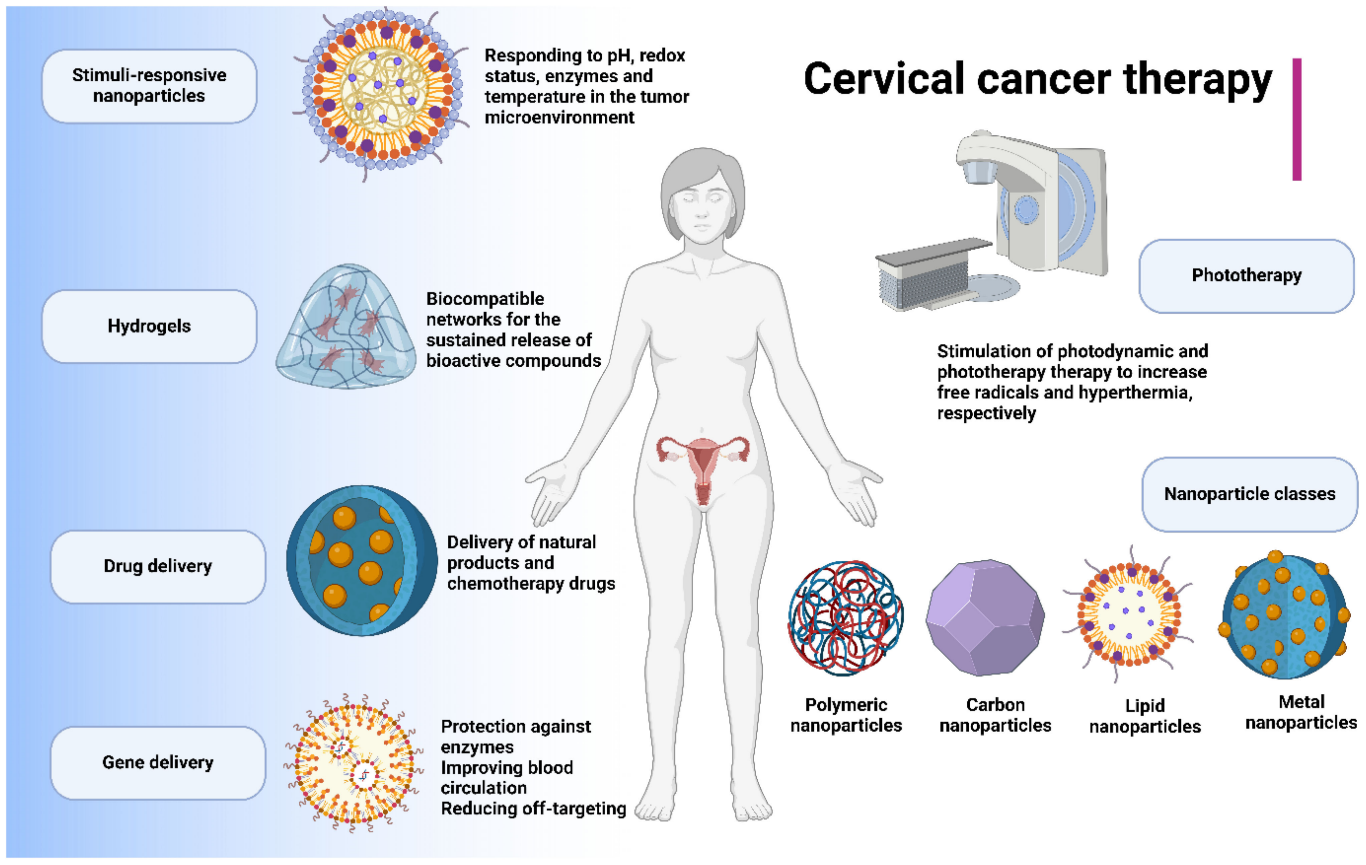
The introduction of nanoparticles for the treatment of cervical cancer is increasing and the reason is the resistance of tumor cells into conventional therapeutics. Moreover, the application of nanoparticles can improve the response of tumor cells to chemotherapy and radiotherapy. Therefore, different kinds of nanocarriers have been introduced for cervical cancer suppression. The stimuli-responsive nanoparticles have improved the fight against cervical cancer through responding to specific stimuli in the tumor microenvironment including pH, redox, enzyme and temperature. Moreover, light-responsive nanoparticles have been utilized for the phototherapy-accelerated tumor ablation. Notably, hydrogels are novel kinds of structures and platforms used for the delivery of bioactive compounds and diagnosis in cervical cancer. Nanoparticles can deliver both drugs and genes to improve the fight against cervical cancer. In spite of the introduction of natural products for the treatment of cervical cancer, these compounds suffer from poor bioavailability and therefore, nanostructures can improve their pharmacokinetic profile in cervical cancer therapy.

Based on the research, targeted drugs like bevacizumab and pembrolizumab have limited efficacy in treating cervical cancer (227). High levels of serum EGF and tissue EGF in cervical cancer patients lead to poor prognosis (228–230). Using hydrogels to lower EGF concentration around tumors is a promising strategy to inhibit tumor growth and recurrence. In this regard, thermo-responsive PLGA-PEG-PLGA hydrogel has been used for continuous delivery of EGF to prevent recurrence of cervical cancer (231). The desirable characteristics of the hydrogel enable it to soak up liquid in the vicinity of tumors, constantly releasing EGF at minimal levels, helping to prevent tumor growth in a mouse model of cervical cancer. Compared to EGF that is free, EGF contained in hydrogel reduces cell migration and proliferation by releasing small amounts of EGF. This helps shield the tumor surroundings and slows down the advancement of cancer, providing a hopeful strategy for preventing the recurrence of cervical cancer (231). According to these studies, the development of hydrogels is promising in cervical cancer therapy. However, more focus should be directed towards the application of hydrogels in cervical cancer immunotherapy and the development of vaccines for the long-term suppression of tumor. **Table 1** summarizes the application of nannocarriers in cervical cancer therapy. **Figure 7** is a summary of the nanoparticles utilized in cervical cancer therapy.

**Table 1:** The application of nanoparticles for the treatment of cervical cancer.

Nanoparticle	Cargo	Remark	Reference
Zinc oxide nanostructures	–	Stimulation of apoptosis Development from Solanim nigrum	(232)
Stimuli-responsive nanoparticles	Cisplatin Paclitaxel	Modification with TMTP1 TME-sensitive feature Improving blood circulation time Prolonged release of cargo	(233)
Lipid nanostructures	siRNA	Loading EPV16 E6/E7-siRNA in nanoparticles and application along with cisplatin cancer synergistic cancer therapy	(76)
Boronated chitosan/alginate nanostructures	Paclitaxel	Size less than 390 nm 98.1-99.8% encapsulation efficiency Loading efficiency of 326.9 - 332.7 μg/mg Favorable mucoadhesive property	(234)
Magnetic mesoporous silica nanostructures	XIAP miR-233	Enhancing apoptosis Promoting radiosensitivity	(235)
Polymeric nanostructures	Imiquimod	Chitosan functionalization Loading into gels Anti-cancer activity and reducing the levels of pro-inflammatory cytokines	(236)
Polymeric carriers	Nano-vascular disrupting agents	Desirable anti-cancer activity	(237)
Lipid-polymer hybrid nanocarriers	Paclitaxel Carboplatin	Functionalization with folate pH-responsive feature Favourable cellular uptake High anti-cancer activity	(42)
Silver nanoparticles	–	Capping the nanoparticles with L-histidine Enhancing ROS levels Mediating apoptosis Causing mitochondrial dysfunction	(238)
Selenium nanostructures	–	The development of nanocarriers from Pseudomonas stutzeri (MH191156) Suppression of angiogenesis and proliferation	(239)
Chitosan nanostructures	TGF-β1	Reducing miR-155 levels and increasing Tim-3 levels to mediate tumor suppression	(240)
Polymeric nanostructures	–	Combination therapy using photodynamic therapy and cold atmospheric plasma	(122)
PLGA nanoparticles	Indocyanine green	Functionalization of nanostructures with hyaluronic acid Size of 200 nm Zeta potential of 33 mV Improving cellular internalization	(241)
Solid lipid nanostructures	5-flourouracil	Improving drug release and cancer therapy	(161)
Selenium nanoparticles	siRNA	Modification of nanoparticles with RGDfC peptide Enhancing ROS levels Triggering mitochondrial dysfunction	(75)

## 8 The role of nanobiotechnology in tumor immunotherapy

Nanobiotechnology demonstrates tremendous potential in the treatment of cervical cancer, especially in the context of tumor immunotherapy (242). Tumor immunotherapy aims to harness the patient’s immune system to recognize and attack cancer cells, and nanotechnology can significantly enhance this process (243). For instance, nanoparticles can be designed to carry tumor antigens, boosting the function of antigen-presenting cells (APCs) and eliciting a potent anti-tumor immune response. These nanoparticles not only protect the antigens from degradation within the body but also facilitate their delivery to lymph nodes, improving T-cell activation efficiency. Additionally, nanobiotechnology can help overcome immunosuppressive factors within the tumor microenvironment (244). For example, nanocarriers can be used to precisely deliver immune checkpoint inhibitors, such as PD-1/PD-L1 inhibitors, directly to the tumor site, thereby reducing systemic side effects and enhancing therapeutic efficacy. Moreover, nanoparticles can carry cytokines like IL-2 or GM-CSF, further strengthening tumor-specific immune responses. Another cutting-edge direction is the development of tumor vaccines using nanotechnology. These vaccines typically contain specific tumor-associated antigens, delivered to the immune system via nanocarriers, thus inducing a specific T-cell response. Studies have shown that nanovaccines can not only prevent tumor development but also provide long-lasting immune protection in case of tumor recurrence (245). Overall, the integration of nanobiotechnology and tumor immunotherapy offers new hope for the treatment of cervical cancer. By enabling precise drug delivery and enhancing immune responses, nanotechnology has the potential to improve treatment outcomes and patient prognosis. Continued research and development in this field will further advance personalized and precision treatment strategies for cervical cancer.

Nanobiotechnology and immune cell exhaustion are intricately linked through the application of nanotechnology in the study, detection, treatment, and prevention of immune cell exhaustion. Immune cell exhaustion is a state where T cells and other immune cells progressively lose their function, typically in the context of chronic infections or cancer (246). Nanotechnology offers high-sensitivity nanosensors for detecting biomarkers associated with immune cell exhaustion, enabling early and precise diagnosis. In treatment, nanotechnology facilitates the development of targeted drug delivery systems that direct medications precisely to exhausted immune cells, enhancing therapeutic efficacy and reducing side effects. Additionally, nanotechnology aids in creating advanced nanovaccines that bolster immune cell function and longevity, preventing exhaustion. Furthermore, nanotechnology supports gene editing and repair through nano-carriers that deliver gene editing tools like CRISPR-Cas9 to correct genetic mutations causing immune cell exhaustion. Certain nanomaterials can modulate immune responses, reducing chronic inflammation and reactivating exhausted T cells. As research tools, nanotechnology combined with single-cell analysis provides insights into the characteristics of immune cells in various exhaustion states, revealing underlying molecular mechanisms. This comprehensive understanding can drive the development of novel diagnostic and therapeutic strategies, ultimately improving outcomes for patients with chronic diseases and cancer.

## 9 Interdisciplinary applications of nanotechnology in cervical cancer treatment: from targeted therapy to smart drug delivery

Nanotechnology in the treatment of cervical cancer showcases significant interdisciplinary potential, merging with various fields to drive advancements and innovations (247). By integrating nanotechnology with molecular biology, nanocarriers can deliver drugs or gene therapy molecules specifically to cancer cell markers, enhancing treatment precision (248, 249). Gene editing tools like CRISPR/Cas9 can also be delivered via nanocarriers to modify drug resistance genes, reversing resistance and inhibiting tumor growth (250). In materials science, the development of smart materials, such as pH-sensitive nanocarriers, allows for drug release in the acidic tumor microenvironment, improving treatment efficacy. Functionalized nanoparticles can be engineered to enhance targeting of specific tumor cells or include fluorescent markers for imaging (251). Chemistry contributes through the design of new nanodrug carriers that improve drug solubility and stability, as well as the synthesis of reactive nanomaterials for photodynamic therapy, generating reactive oxygen species to kill cancer cells (252, 253). In medical imaging, nanoprobes enhance tumor detection and localization through improved contrast in MRI, CT, or ultrasound imaging, and real-time monitoring of drug distribution allows for dynamic treatment adjustments (254, 255). Nanotechnology’s intersection with immunology enables the development of nanocarriers for delivering immunoadjuvants, vaccines, or immune checkpoint inhibitors, boosting the body’s immune response to tumors. Targeted delivery to immune cells like T cells can enhance their activity in tumors, improving immunotherapy outcomes. Lastly, pharmacokinetics research benefits from controlled drug release systems developed through nanotechnology, optimizing drug bioavailability and half-life. Overall, the fusion of nanotechnology with these diverse disciplines enhances treatment strategies for cervical cancer, fostering innovation and offering new hope for patients.

## 10 Evaluating preclinical models for nanotechnology therapies: a comparative study of *in vitro* systems, animal models, and organ-on-a-chip technologies

Preclinical models play a crucial role in evaluating nanotechnology approaches for cervical cancer treatment, encompassing *in vitro* cell culture systems, animal models, and organ-on-a-chip technologies (256). *In vitro* cell culture systems, including monolayer and three-dimensional (3D) cultures, provide foundational insights into drug effects on cells, mimicking basic tumor cell characteristics such as proliferation and drug responses (257). However, they often fall short in replicating the complex human physiological environment, leading to potential discrepancies between *in vitro* and *in vivo* results. Animal models, such as xenograft and spontaneous tumor models, offer a more comprehensive view by simulating tumor growth, metastasis, and systemic drug metabolism within a whole organism (258, 259). Despite their closer alignment with human physiology, species differences in immune responses and metabolic pathways can limit their predictive accuracy for clinical outcomes. Organ-on-a-chip technologies represent a significant advancement by creating miniaturized organ models that simulate human physiological functions and interactions with higher fidelity (260, 261). These models address some limitations of traditional methods by providing a more realistic drug response environment but still face challenges in fully replicating the complexity of human biology and achieving standardization. Integrating these models allows for a more holistic assessment of nanotechnology therapies, enhancing the prediction of clinical potential and identifying possible challenges.

## 11 Conclusion and clinical perspectives

The cervical cancer is one of the leading causes of death worldwide. In spite of the using various kinds of therapeutics for the treatment of cervical cancer, this tumor is still causing high death among patients. This is mainly due to the late diagnosis of cancer patients and also, the development of therapy resistance. As a result, the nanoparticles have been introduced as alternative factors in cervical cancer therapy. The present review demonstrated that nanoparticles are potential factors for the treatment of cervical cancer through the delivery of natural products and chemotherapy drugs. For the natural products, they have poor bioavailability and the application of nanostructures can improve their pharmacokinetic profile in cervical cancer therapy. In case of chemotherapy drugs, the nanoparticles can enhance the cellular internalization to reverse drug resistance and enhance the cytotoxicity. Moreover, the nanoparticles can co-deliver natural products and chemotherapy drugs. Notably, the genes suffer from degradation by enzymes and off-targeting feature, providing the rational reason for the application of nanoparticles in their delivery in cervical cancer suppression. The nanoparticles can cause phototherapy (PTT and PDT) to cause tumor ablation through mediating hyperthermia and enhancing ROS generation. The stimuli-responsive nanocarriers have improved the potential in cervical cancer therapy through responding to pH, redox, temperature and enzymes. Overall, different kinds of nanocarriers have been developed for the treatment of cervical cancer in which lipid nanostructures are of importance due to their favourable biocompatibility and biosafety. Although the main focus of current paper is on nanoparticles, the hydrogels have been recently applied in the treatment of cervical cancer and these polymeric networks can provide the sustained release of therapeutics along with diagnostic application.
